# Comparative analysis of xenobiotic metabolising *N*-acetyltransferases from ten non-human primates as *in vitro* models of human homologues

**DOI:** 10.1038/s41598-018-28094-6

**Published:** 2018-06-27

**Authors:** Theodora Tsirka, Maria Konstantopoulou, Audrey Sabbagh, Brigitte Crouau-Roy, Ali Ryan, Edith Sim, Sotiria Boukouvala, Giannoulis Fakis

**Affiliations:** 1 Democritus University of Thrace, Department of Molecular Biology and Genetics, Alexandroupolis, Greece; 2 IRD UMR216, Mère et enfant face aux infections tropicales, Paris, France; 3 PRES Sorbonne Paris Cité, Université Paris Descartes, Faculté de Pharmacie, Paris, France; 4 CNRS, Université Paul Sabatier, ENFA, UMR5174EDB (Laboratoire Évolution & Diversité Biologique), Toulouse, France; 5 Université de Toulouse 3, UMR5174EDB Toulouse, France; 6 University of Oxford, Department of Pharmacology, Oxford, UK; 7 Kingston University London, Faculty of Science, Computing and Engineering, Kingston upon Thames, UK

## Abstract

Xenobiotic metabolising *N*-acetyltransferases (NATs) perform biotransformation of drugs and carcinogens. Human NAT1 is associated with endogenous metabolic pathways of cells and is a candidate drug target for cancer. Human NAT2 is a well-characterised polymorphic xenobiotic metabolising enzyme, modulating susceptibility to drug-induced toxicity. Human NATs are difficult to express to high purification yields, complicating large-scale production for high-throughput screens or use in sophisticated enzymology assays and crystallography. We undertake comparative functional investigation of the NAT homologues of ten non-human primates, to characterise their properties and evaluate their suitability as models of human NATs. Considering the amount of generated recombinant protein, the enzymatic activity and thermal stability, the NAT homologues of non-human primates are demonstrated to be a much more effective resource for *in vitro* studies compared with human NATs. Certain NAT homologues are proposed as better models, such as the NAT1 of macaques *Macaca mulatta* and *M*. *sylvanus*, the NAT2 of *Erythrocebus patas*, and both NAT proteins of the gibbon *Nomascus gabriellae* which show highest homology to human NATs. This comparative investigation will facilitate *in vitro* screens towards discovery and optimisation of candidate pharmaceutical compounds for human NAT isoenzymes, while enabling better understanding of NAT function and evolution in primates.

## Introduction

The xenobiotic metabolising *N*-acetyltransferase (NAT) enzymes are involved in the biotransformation of drugs and carcinogens. NATs are conjugation enzymes typically employing acetyl-coenzyme A (CoA) as donor substrate to perform *N*-acetylation of an arylamine or arylhydrazine acceptor substrate during a two-step ping-pong Bi-Bi reaction catalysed by a triad of cysteine, histidine and aspartate residues^1,2^. The diversity of NAT homologues is extensive, as evidenced through previous comparative genomic surveys and phylogenetic analyses across a broad taxonomic range of prokaryotes and eukaryotes^3–6^. The phylogeny of *NAT* homologues in both simple (fungi) and complex (vertebrates) eukaryotes suggests a dynamic birth-and-death evolutionary pattern, where multiple lineage-specific gene duplications and losses, combined with selection-driven sequence divergence, have promoted functional diversification of NAT enzymes, presumably allowing rapid adaptation to variable xenobiotic environments^5,6^. Enzymatic and structure-function investigations, particularly with microbial NATs, support this model, as they reveal variability even in key catalytic components in certain organisms^7–10^.

In primates, our phylogenetic analysis^6^ predicted an initial duplication of the ancestral *NAT* gene in the common ancestor of Simiiformes, generating *NAT1* and a second *NAT* gene which subsequently duplicated in the common ancestor of Catarrhini (Old World monkeys and apes, including human) to give rise to *NAT2* and the pseudogene *NATP*. Platyrrhini (New World monkeys) appear to lack the third *NAT* locus, while other independent duplication events may have occurred in the lower primate lineages of Strepsirrhini and Tarsiiformes. NAT1 evolution in primates appears to be primarily subject to purifying selection, potentially maintaining some endogenous metabolic function. NAT2 evolution is more likely driven by diversifying selection favouring variability in amino acid sequence, possibly as adaptation to changing xenobiotic exposures. These observations are supported by population genetic studies of human *NAT* genes, predicting that changing selective pressures on NAT2, rather than NAT1, may have favoured expansion of the slow acetylator genotype/phenotype after the agricultural revolution of the Neolithic period^11–16^. Human *NAT1* gene expression begins very early in embryogenesis^17^ and is evident in a range of tissues, driven by a housekeeping promoter^18,19^. By contrast, human *NAT2* shows a more restricted expression pattern consistent with its well-established role as hepatic xenobiotic metabolising enzyme^20^. Polymorphisms are also more common and their effects on xenobiotic acetylation more apparent for human *NAT2* compared with *NAT1* gene^21^.

Expanding previous *in silico* phylogenetic analyses of primate NATs^6^, we undertook a comparative functional investigation of the NAT1 and NAT2 homologues of eleven primate species, including human. The recombinant NAT proteins of non-human primates are proposed as models of their human counterparts, particularly for *in vitro* screens towards the discovery and optimisation of candidate pharmaceutical compounds.

## Results and Discussion

### Primate *NAT* genes and proteins

We have previously reported^6^ sequencing of *NAT* genes from the non-human primates *Sapajus apella* (a New World monkey from South America), *Allenopithecus nigroviridis*, *Cercopithecus diana*, *Chlorocebus tantalus*, *Erythrocebus patas*, *Macaca sylvanus* and *Mandrillus sphinx* (Old World monkeys from across Africa), *Macaca mulatta* and *Trachypithecus cristatus* (Old World monkeys from South Asia), and *Nomascus gabriellae* (an ape from Southeast Asia). Of those ten species, the gibbon *N*. *gabriellae* is taxonomically more closely related to *Homo sapiens* (human). The corresponding consensus classification of these primates is shown in Supplementary Fig. S1, with additional information provided in Supplementary Table S1.

Suitable species-specific mnemonics were assigned to symbols of annotated *NAT* genes (Table 1), according to current consensus nomenclature rules^22^, and complete coding sequences of 873 bp were cloned in expression vector. The translated 290 amino acid NAT sequences were highly conserved; identity ranged from 94.16 to 100% for NAT1 and from 85.57 to 99.66% for NAT2 sequences, with higher variability observed among NAT2 than among NAT1 homologues (Supplementary Fig. S2 and Supplementary Table S2). The phylogeny of protein sequences was in line with the species taxonomy of Supplementary Fig. S1, but the NAT1 and NAT2 homologues clustered as separate groups, supporting two distinct lineages of *NAT* orthologues in the simians (Supplementary Fig. S3). These observations are consistent with our earlier evolutionary studies of primate NATs^6^.

**Table 1:** Description of *NAT* gene constructs and their primate species of origin.

Scientific name (synonym)^a^	Common name^a^	Taxon mnemonic^a^	Taxon ID^a^	Gene^b^	GenBank ID^b^	Amplification primers^c^
*Allenopithecus nigroviridis*	Allen’s swamp monkey	ALLNI	54135	*NAT1*	KU640972	#1 and #2
*Cercopithecus diana*	Diana monkey	CERDI	36224	*NAT1*	KU640973	#1 and #2
				*NAT2*	KU640982	#1 and #3
*Chlorocebus tantalus* (*Cercopithecus tantalus*)	Tantalus monkey	CHLTN	60712	*NAT1*	KU640974	#6 and #7
				*NAT2*	KU640983	#1 and #3
*Erythrocebus patas* (*Cercopithecus patas*)	Red guenon	ERYPA	9538	*NAT1*	KU640975	#6 and #7
				*NAT2*	KU640984	#1 and #3
*Homo sapiens*	Human	HUMAN	9606	*NAT1*	X17059 (allele NAT1*4)	#1 and #2
				*NAT2*	X14672 (allele NAT2*4)	Clone available
*Macaca mulatta*	Rhesus macaque	MACMU	9544	*NAT1*	KU640969	#1 and #2
				*NAT2*	AJ504440 (allele NAT2*1)	Clone available
*Macaca sylvanus*	Barbary macaque	MACSY	9546	*NAT1*	KU640970	#1 and #2
				*NAT2*	KU640978	#1 and #3
*Mandrillus sphinx* (*Papio sphinx*)	Mandrill	MANSP	9561	*NAT1*	KU640971	#1 and #2
				*NAT2*	KU640980	#1 and #3
*Nomascus gabriellae*	Red-cheeked gibbon	NOMGA	61852	*NAT1*	KU640967	#1 and #2
				*NAT2*	KU640985	#1 and #4
*Sapajus paella* (*Cebus apella*)	Brown-capped capuchin	SAPAP	9515	*NAT1*	KU640966	#1 and #2
*Trachypithecus cristatus* (*Presbytis cristata*)	Silvered leaf-monkey	TRACR	122765	*NAT1*	KU640968	#1 and #2
				*NAT2*	KU640977	#1 and #5

^a^The current scientific names of species with synonymous names in parentheses (first column) and a common name (second column), are from the UniProt Taxonomy database (http://www.uniprot.org/taxonomy/). Official taxon mnemonics (third column) and taxon identification numbers (fourth column) are from the same source. According to current consensus nomenclature (http://nat.mbg.duth.gr/), taxon mnemonics are attached to the symbols of *NAT* genes to identify their specific organism of origin^22^.

^b^All *NAT* gene annotations (fifth column) are as first described by^6^, except for the *NAT2* gene of *M*. *mulatta*^60^, and the *NAT1* and *NAT2* genes of *H*. *sapiens*^66^. The recombinant proteins expressed in this study were as predicted by translation of the nucleotide sequences for which GenBank identification numbers are provided (sixth column).

^c^The complete coding sequence of each *NAT* gene was amplified for cloning, using combinations of the following primers, as indicated (seventh column): Primer #1 (forward), GCGGCAGCCATATGGACATTGAAGCATA; Primer #2 (reverse), TCGAGTGCGGCCGCCTAAATAGTAAAAAATCTATCACC; Primer #3 (reverse), GTGCTCGAGTGCGGCCGCCTAAATAGTGAAGGATC; Primer #4 (reverse), TCGAGTGCGGCCGCCTAAATAGTAAGGGATCCATC; Primer #5 (reverse), TCGAGTGCGGCCGCCTAAATAGTAAAGAATCCATC. The recognition sites for restriction endonucleases *Nde*I (forward primer) and *Not*I (reverse primers) are underlined. Clones for the *NAT2* genes of *M*. *mulatta* and *H*. *sapiens* were available in our laboratories from previous studies^61^. The *NAT1* constructs of *C*. *tantalus* and *E*. *patas* were generated from the *NAT1* construct of *C*. *diana* via site-directed mutagenesis with Primer #6 (forward), CCATGGACTTAG**G**CTTAGAGGCCAT, and Primer #7 (reverse), ATGGCCTCTAAG**C**CTAAGTCCATGG (in bold, the mutagenised nucleotide representing the only non-synonymous SNP differentiating the *NAT1* gene of *C*. *diana* from the other two homologues). Amplification of the *NAT2* genes of *A*. *nigroviridis* (GenBank ID: KU640981) and *S*. *apella* (GeneBank ID: KU640976) was not possible due to limited availability of genomic DNA.

**Figure 1:**
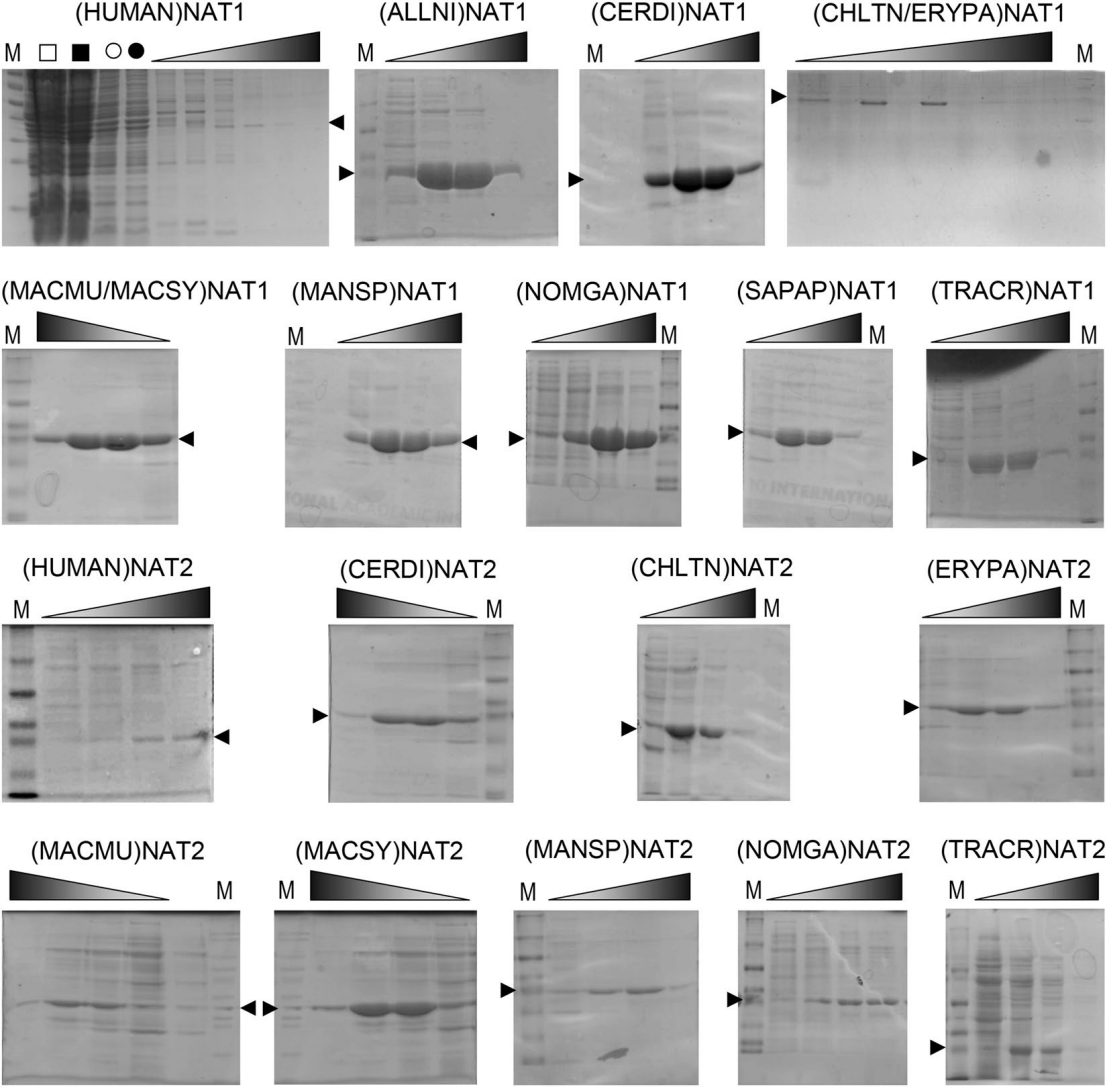
Recombinant NAT proteins of primates. The NAT proteins of *Homo sapiens* (HUMAN), *Allenopithecus nigroviridis* (ALLNI), *Cercopithecus diana* (CERDI), *Chlorocebus tantalus* (CHLTN), *Erythrocebus patas* (ERYPA), *Macaca mulatta* (MACMU), *Macaca sylvanus* (MACSY), *Mandrillus sphinx* (MANSP), *Nomascus gabriellae* (NOMGA), *Sapajus apella* (SAPAP) and *Trachypithecus cristatus* (TRACR) were expressed in *E. coli* and isolated by affinity chromatography. *C. tantalus* and *E. patas* have identical NAT1 amino acid sequences, thus only one recombinant protein was expressed, designated (CHLTN/ERYPA)NAT1. This was also the case for the identical NAT1 proteins of *M. mulatta* and *M. sylvanus*, expressed as (MACMU/MACSY)NAT1. SDS-PAGE gels are shown with chromatographic fractions eluted with a concentration gradient of up to 250 mM of imidazole, indicated by shaded triangles at the top of each image. Each lane was loaded with 30 μl of eluate. The symbols in the first image are whole cell lysate without IPTG induction (□), whole cell lysate after IPTG induction (■), soluble cell extract before the affinity chromatography (○), and initial flowthrough of soluble cell extract through the affinity chromatography column (●). The small black arrowheads indicate bands of recombinant NAT proteins, the molecular weight of which (~31 kDa) was estimated relative to protein markers (lanes M). Full-length gels are presented in Expanded Data Supplementary Fig. 1 at the end of the Supplementary Information file.

Nine NAT1 homologues were expressed in recombinant form, representing all eleven primate species in this study, including human. NAT1 protein sequences were identical between *C*. *tantalus* and *E*. *patas*, as well as between *M*. *mulatta* and *M*. *sylvanus*. Expression of nine NAT2 homologues was also performed, representing all species except *A*. *nigroviridis* and *S*. *apella* (Table 1). Using a standard expression and affinity chromatography purification method, the 18 recombinant proteins were recovered at variable levels of yield and purity (Fig. 1). Expression of NAT homologues of non-human primates was consistently higher compared with (HUMAN)NAT1 and (HUMAN)NAT2 proteins, which were generated in very low amounts. Overall, the expression of non-human NAT1 homologues was considerably higher compared with non-human NAT2 homologues, enabling more effective purification of the former.

Unlike the (HUMAN)NAT proteins, further chromatographic purification was possible for the gibbon (NOMGA)NAT1 and (NOMGA)NAT2, as well as for other readily expressed NAT homologues of non-human primates. Under the gel filtration conditions used, the NAT proteins were recovered as monomers with the expected molecular mass (Supplementary Fig. S4). Purified proteins (ALLNI)NAT1, (NOMGA)NAT1 and (TRACR)NAT1 (Supplementary Fig. S5) were further selected for crystallisation screens with commercial panels (Morpheus^®^, JCSG plus^TM^, Midas^TM^, Structure Screen 1 & 2 HT-96 and Wizard, all from Molecular Dimensions), using the facilities of the Department of Biochemistry at Oxford University. Although these initial crystallisation attempts did not provide any useful crystals, the production of purified NAT proteins (3–10 mg per litre of culture) from various non-human primates is encouraging, laying the groundwork for future screens.

Previous studies have shown that human NATs are very difficult to express to high purification yields in *Escherichia coli*, complicating their large-scale production for high-throughput screens of chemical libraries, or for use in sophisticated crystallographic and enzymological investigations. For crystallisation, Wu and colleagues^23^ generated recombinant (HUMAN)NAT1 and (HUMAN)NAT2 proteins to purification yields of 1.6 and 0.5 mg per litre of bacterial culture, respectively (http://www.thesgc.org/structures/2IJA; http://www.thesgc.org/structures/2PQT; http://www.thesgc.org/structures/2PFR). Another group has also been successful in producing fully purified (HUMAN)NAT1 and (HUMAN)NAT2 proteins to high yields (4 and 1.4 mg/L, respectively), and these preparations have been used to advance knowledge of NAT enzymatic catalysis and inhibition^24–27^. However, the bulk of studies measuring recombinant activity of human NAT isoenzymes have employed either crude lysates (of bacterial, yeast or mammalian cells) or partially purified proteins in low yields. In many instances, enzymatic activity of human NATs and their polymorphic variants has been measured in preparations where recombinant protein is detectable only by Western blot^28–40^.

The recombinant NAT proteins of laboratory rodents have been popular substitutes of human NATs in studies of catalytic function or *in vitro* screens for pharmaceutical compounds^39–47^. However, compared with the NAT proteins of non-human primates, the rodent homologues are less suitable models, given their considerably lower identity to human NATs (Supplementary Table S2). Moreover, although human *NAT1* and rodent *Nat2* genes are orthologous (i.e. they share common phylogenetic ancestry and function), this is not true for human *NAT2* and rodent *Nat1* genes which have derived through independent genomic duplications occuring after the divergence of rodents and primates^6,44,47^.

The advantages of expressing recombinant NAT1 and NAT2 proteins of non-human primates, for use as *in vitro* models of their human homologues, are obvious. In particular, the gibbon *N*. *gabriellae* could serve as a convenient model, since it was possible to efficiently express and purify its NAT isoenzymes (Supplementary Fig. S4 and Supplementary Fig. S5). Sequence identity is 95.88% between (HUMAN)NAT1 and (NOMGA)NAT1, as well as between (HUMAN)NAT2 and (NOMGA)NAT2, which corresponds to a difference of only 12 amino acids per each pair of homologues (Supplementary Fig. S2 and Supplementary Table S2).

### Enzymatic activities of primate NAT proteins

**Figure 2:**
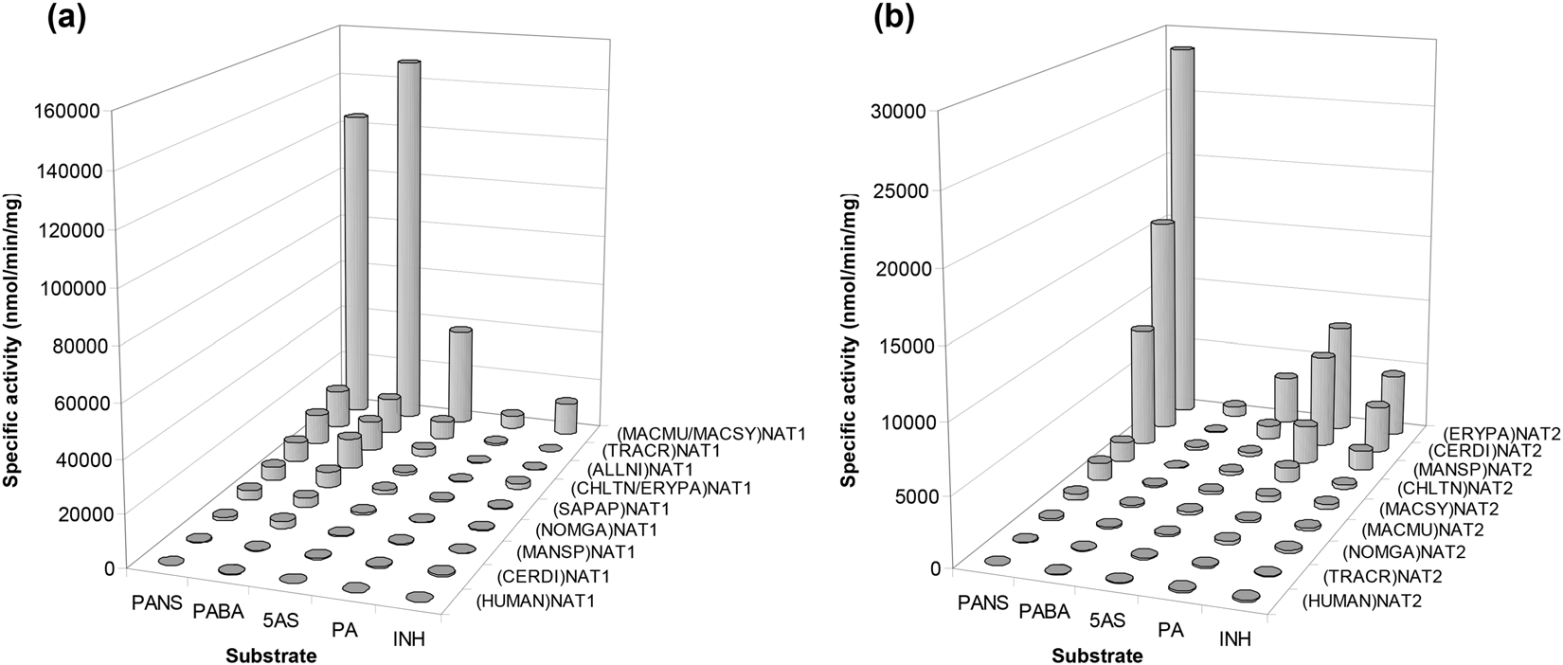
Enzyme activity screens of primate NAT homologues. Overview of the specific activities measured with the recombinant NAT1 (**a**) and NAT2 (**b**) isoenzymes of primate species *Allenopithecus nigroviridis* (ALLNI), *Cercopithecus diana* (CERDI), *Chlorocebus tantalus* (CHLTN), *Erythrocebus patas* (ERYPA), *Macaca mulatta* (MACMU), *Macaca sylvanus* (MACSY), *Mandrillus sphinx* (MANSP), *Nomascus gabriellae* (NOMGA), *Sapajus apella* (SAPAP), *Trachypithecus cristatus* (TRACR) and *Homo sapiens* (HUMAN). Assays were performed with acetyl-CoA as acyl-group donor substrate and a series of acceptor substrates, namely *p*-anisidine (PANS), *p*-aminobenzoate (PABA), 5-aminosalicylate (5AS), procainamide (PA) and isoniazid (INH). Enzyme activity was detected with all NAT homologues, but varied considerably per protein and substrate used. Assays were performed in duplicate and variability between replicates was within 10% from the average specific activity shown. A presentation of enzyme activity data is provided in Expanded Data Supplementary Fig. 2 at the end of the Supplementary Information file.

All recombinant NAT proteins were assayed for enzymatic activity against the (HUMAN)NAT1 selective substrate *p*-aminobenzoate (PABA) and the (HUMAN)NAT2 selective substrates procainamide (PA, an antiarrhythmic arylamine) and isoniazid (INH, an anti-tubercular arylhydrazine). Assays were also performed with the less selective arylamine substrates 5-aminosalicylate (5AS) and *p*-anisidine (PANS)^35^. Overall, the NAT isoenzymes of non-human primates demonstrated the expected substrate specificity pattern, but enzyme activities varied considerably among species (Fig. 2).

The specific activities measured for (HUMAN)NAT1 and (HUMAN)NAT2 were lowest, even with selective substrates, and this was possibly due to the poor quality of recombinant preparations generated for those two proteins under the standard expression/purification conditions used (Fig. 1). However, the gibbon (NOMGA)NAT1 and (NOMGA)NAT2 isoenzymes provided relatively low specific activities too, despite their more robust recombinant expression and purification. Compared with other primates, NAT enzymatic activities may be lower in human and other apes. This similarity, further supports the utility of (NOMGA)NAT1 and (NOMGA)NAT2 as *in vitro* models of the more poorly expressed human NATs.

The non-selective substrate PANS was efficient with both the NAT1 and the NAT2 isoenzymes of non-human primates (Fig. 2). The (MACMU/MACSY)NAT1 homologue of the two macaque species generated excessive activities with both PANS and PABA, which were at least 8-fold higher than the activities measured for all other NAT1 homologues (Fig. 2a). Similarly, (ERYPA)NAT2 was the most active NAT2 isoenzyme with PANS and its selective substrates PA and INH (Fig. 2b).

**Figure 3:**
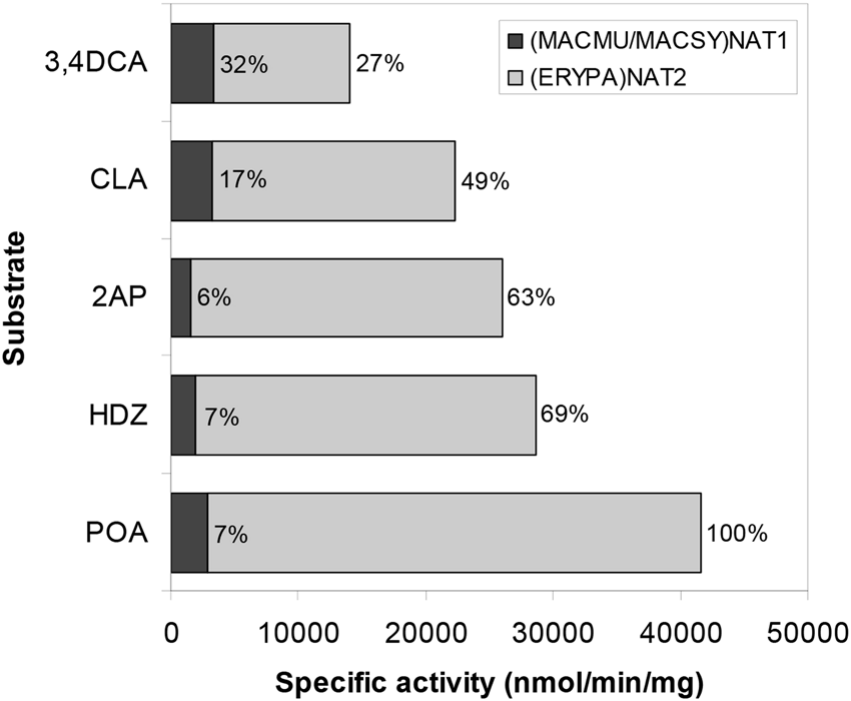
Additional acceptor substrate preferences of primate NAT1 and NAT2 homologues. The arylamines 4-phenoxyaniline (POA), 2-aminophenol (2AP), 4-chloroaniline (CLA) and 3,4-dichloroaniline (3,4DCA), and the arylhydrazine hydralazine (HDZ) were used in assays with either (MACMU/MACSY)NAT1 of the two macaques or (ERYPA)NAT2 of *Erythrocebus patas*. These were the recombinant NAT proteins providing highest activities in the screens of Fig. 2. In the plot, the light grey bars are the specific activities of (ERYPA)NAT2 with each one of the five substrates tested, also provided as percentages (%) relative to the specific activity measured with POA (100%). The dark grey bars are the specific activities of (MACMU/MACSY)NAT1, and each measurement is also provided relative (%) to the corresponding specific activity of (ERYPA)NAT2 per each substrate tested. Assays were performed in duplicate and variability between replicates was within 10% from the average specific activity shown. A presentation of enzyme activity data is provided in Expanded Data Supplementary Fig. 2 at the end of the Supplementary Information file.

The highly active (MACMU/MACSY)NAT1 and (ERYPA)NAT2 homologues were further used to test selectivity against a series of additional NAT substrates, namely the toxic arylamines 2-aminophenol (2AP), 4-chloroaniline (CLA), 3,4-dichloroaniline (3,4DCA) and 4-phenoxyaniline (POA), and the pharmaceutical arylhydrazine hydralazine (HDZ)^35^. Both NAT homologues provided activity with all five substrates, but the activities measured with (ERYPA)NAT2 were substantially higher (>10,000 nmol/min/mg) compared with those measured with (MACMU/MACSY)NAT1 (<3500 nmol/min/mg) (Fig. 3). The results suggest that 2AP, CLA, 3,4DCA, POA, and HDZ are more NAT2 selective, particularly taking into account that (MACMU/MACSY)NAT1 was about 4.5-fold more active than (ERYPA)NAT2 with the non-selective substrate PANS (Fig. 2). The pattern agrees with previous investigations with human NATs, generated in low yields detectable by Western blot^35^.

**Figure 4:**
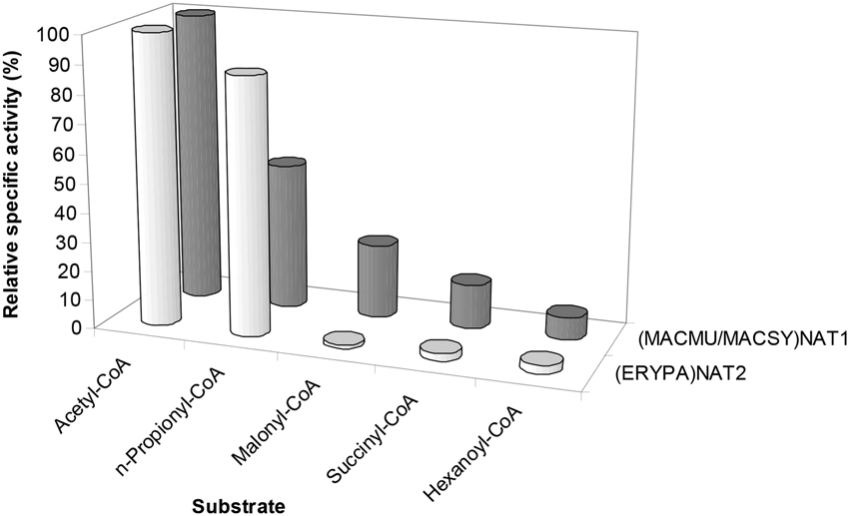
Donor substrate preferences of primate NAT1 and NAT2 homologues. Two parallel sets of assays were performed with acetyl-, *n*-propionyl-, malonyl-, succinyl- or hexanoyl-CoA as donor substrates. The first set employed (MACMU/MACSY)NAT1 protein with *p*-aminobenzoate (PABA) as acceptor substrate, while the second utilised (ERYPA)NAT2 protein with *p*-anisidine (PANS). On the graph, the bars of each series are the relative specific activities measured with each one of the five acyl-group donor substrates tested, presented as percentage (%) of the corresponding specific activity measured with acetyl-CoA (100%). Assays were performed in triplicate and variability between replicates was within 10% from the calculated average specific activity. A presentation of enzyme activity data is provided in Expanded Data Supplementary Fig. 2 at the end of the Supplementary Information file.

Previous studies have demonstrated that NATs can utilise acyl-group donor substrates other than acetyl-CoA^10,35,48^. We thus tested (MACMU/MACSY)NAT1 and (ERYPA)NAT2 with acetyl-, *n*-propionyl-, malonyl-, succinyl- and hexanoyl-CoA as donor substrates. As demonstrated (Fig. 4), (MACMU/MACSY)NAT1 provided activities with all five acyl-CoA compounds, in the following declining order: acetyl-CoA (100%) > *n*-propionyl-CoA (50%) > malonyl-CoA (25%) > succinyl-CoA (15%) and hexanoyl-CoA (8%). Activities with these compounds have also been reported with hamster NAT2 recombinant protein, which is functionally equivalent to human NAT1^35^. The (ERYPA)NAT2 protein demonstrated more restricted acyl-CoA selectivity, utilising acetyl-CoA (100%) and *n*-propionyl-CoA (88%), but providing only marginal (<3%) activities with the remaining three acyl-CoA substrates tested. This pattern is more similar to that observed with certain microbial NATs, which are effective utilisers of acetyl- and *n*-propionyl-CoA, but show virtually no preference for other acyl-CoA compounds^10,48^.

### Thermal stability of primate NAT proteins

**Figure 5:**
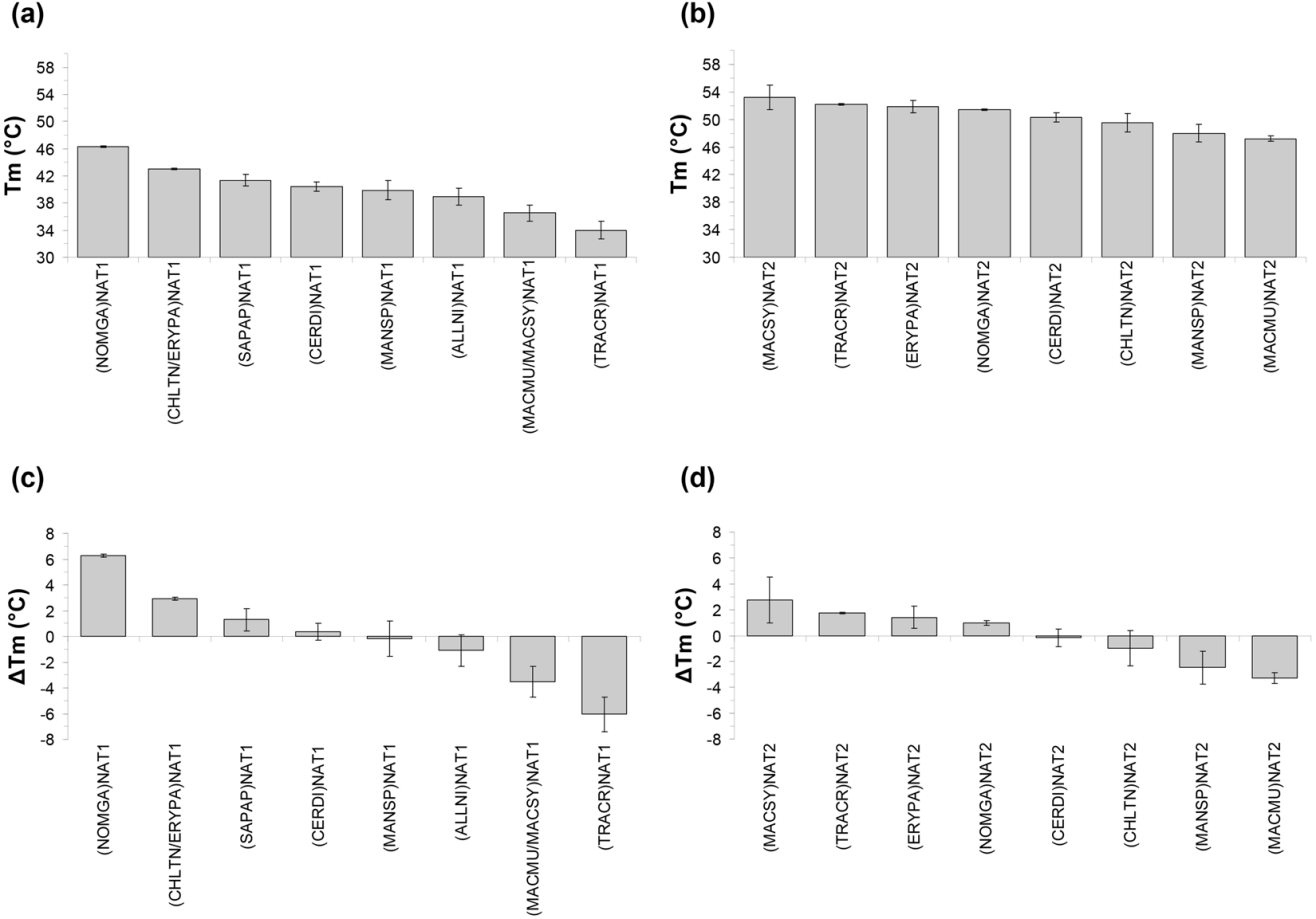
Thermal stability of primate NAT proteins. Overview of Tm values determined by DSF analysis of recombinant NAT1 (**a**) and NAT2 (**b**) homologues of non-human primates *Allenopithecus nigroviridis* (ALLNI), *Cercopithecus diana* (CERDI), *Chlorocebus tantalus* (CHLTN), *Erythrocebus patas* (ERYPA), *Macaca mulatta* (MACMU), *Macaca sylvanus* (MACSY), *Mandrillus sphinx* (MANSP), *Nomascus gabriellae* (NOMGA), *Sapajus apella* (SAPAP) and *Trachypithecus cristatus* (TRACR). The difference of each NAT1 (**c**) or NAT2 (**d**) Tm value from the corresponding calculated mean temperature is also plotted (the *x*-axis of graphs c and d corresponds to mean values of 40.07 °C and 50.45 °C for NAT1 and NAT2 proteins, respectively). Duplicate experiments were performed generating sigmoid curves that were fitted to the Boltzmann equation (Adj. R^2^ ≥ 0.99). The screens were typically repeated multiple times with different protein preparations (particularly for low-Tm NAT homologues), in order to ensure accuracy and reproducibility of results.

Differential Scanning Fluorimetry (DSF) is widely employed as a quick, sensitive, low-cost, high-throughput technique for initial screens of pharmaceutical targets against candidate inhibitors or other ligands^49,50^. We found that application of the technique with (HUMAN)NAT1 and (HUMAN)NAT2 is complicated by the poor quality of recombinant protein preparations generated with our expression/purification scheme, which is commonly used by many laboratories. In contrast, DSF was successful with the recombinant NAT proteins of non-human primates, allowing accurate assessment of their thermal stability with or without substrates (Figs 5 and S6). The use of recombinant NATs from non-human primates would, therefore, facilitate initial DSF-based screens of chemical libraries for identification of leads that may be further evaluated as potential inhibitors of the human homologues. For example, the gibbon (NOMGA)NAT1 and (NOMGA)NAT2 isoenzymes appeared quite robust in DSF assays, providing Tm values of 46.34 ± 0.11 and 51.42 ± 0.15 °C, respectively. As mentioned above, the gibbon NAT proteins are highly similar in amino acid sequence with their human homologues (Supplementary Table S2) and they are readily expressed and purified in recombinant form (Supplementary Fig. S4). Both (NOMGA)NAT isoenzymes provided Tm values in the higher range (Fig. 5), generating optimal DSF curves. The recombinant NAT homologues of the gibbon are, therefore, proposed as appropriate models of human NATs in DSF-based screens for candidate pharmaceutical compounds.

The determined Tm values for NAT homologues of non-human primates ranged from 34.01 ± 1.34 to 46.34 ± 0.11 °C (mean value 40.07 ± 3.78 °C) for NAT1, and from 47.18 ± 0.4 to 53.23 ± 1.77 °C (mean value 50.45 ± 2.12 °C) for NAT2, supporting that the NAT2 homologues are considerably more resilient to thermal denaturation than the NAT1 homologues, as has been reported previously for the human NAT isoenzymes^31^. Among NAT1 proteins, (NOMGA)NAT1 and (TRACR)NAT1 had the highest and lowest Tm values, respectively, translating into a difference of about 12 °C (Fig. 5a and c). Variability in Tm was more restricted among NAT2 proteins (maximum difference of about 6 °C), with (MACSY)NAT2 and (MACMU)NAT2 providing the highest and lowest Tm values, respectively (Fig. 5b and d). This is noteworthy, since those two proteins are 99.31% identical and belong to species of the same genus (Supplementary Fig. S2 and Supplementary Table S2).

### Other comparative aspects of primate NAT1 homologues

Human NAT1 has been the focus of investigations seeking to unravel its postulated role in endogenous metabolism and disease, particularly cancer^51–53^. Human NAT1 and its murine homologue have been implicated in folate metabolism via the acetylation of *p*-aminobenzoylglutamate (pABGlu)^51^, although recent studies suggest a more complex association between folate and NAT^40,54,55^. We show here that NAT1 is capable of pABGlu acetylation in non-human primates too, as expected (Supplementary Fig. S7).

**Figure 6:**
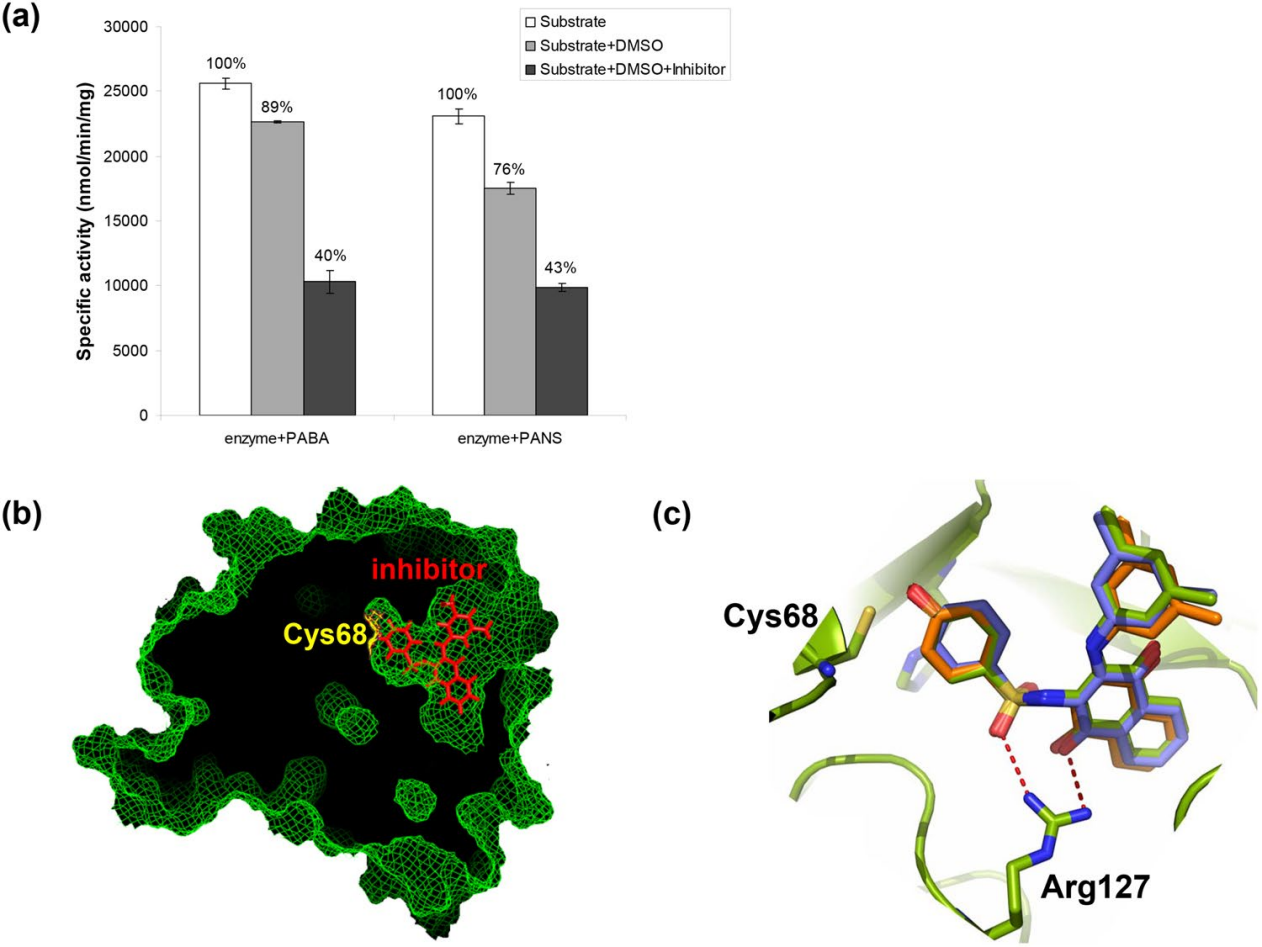
Interaction of primate NAT1 homologues with a small-molecule inhibitor. A synthetic naphthoquinone inhibitor of (HUMAN)NAT1 and (MOUSE)NAT2^46^ was used. (**a**) Inhibition of (MACMU/MACSY)NAT1 homologue by 10 μM of the inhibitor in 5% (v/v) DMSO. Two replicate experiments were performed and the results are provided as average specific activity ± standard deviation. The enzymatic substrates used were *p*-aminobenzoate (PABA) and *p*-anisidine (PANS). (**b**) Overview of inhibitor binding to the active site of (MACMU/MACSY)NAT1 protein, modelled against the crystal structure of (HUMAN)NAT1 (PDB ID: 2PQT). (**c**) Detailed view of inhibitor binding to the superimposed models of (HUMAN)NAT1, (NOMGA)NAT1 and (MACMU/MACSY)NAT1 proteins. A slight shift in positioning of the inhibitor (particularly its *m*-xylene ring) within the active site of (MACMU/MACSY)NAT1 was observed, relative to the other two NAT1 homologues where binding showed complete overlap.

Moreover, human NAT1 has been investigated as a candidate target for small-molecule inhibition^56^. A naphthoquinone inhibitor developed for (HUMAN)NAT1^46^, also inhibited (MACMU/MACSY)NAT1 at 10 μM concentration (Fig. 6a). However, inhibition was lower (50% decrease in enzyme activity with PABA) for (MACMU/MACSY)NAT1, compared with the reported 92% with 5AS for (HUMAN)NAT1^46^. Our enzymatic assays (Fig. 2a) show that (MACMU/MACSY)NAT1 is much more active than (HUMAN)NAT1, indicating that full inhibition would likely require a higher concentration of the naphthoquinone in the reaction. The *in silico* docking of the inhibitor to the modelled structure of (MACMU/MACSY)NAT1 protein indicated only a minor shift to the expected positioning within the active site. Docking of the inhibitor was the same between (HUMAN)NAT1 and the modelled structure of (NOMGA)NAT1 (Fig. 6b,c). The limited availability of the compound at the time of this study precluded more sophisticated assays using a concentration range of the inhibitor with NAT1 proteins from different non-human primates. Since those efficiently expressed homologues may constitute a useful resource for future high-throughput chemical screens, further optimisation of inhibition assays is warranted. DSF would also be useful as a more affordable high-throughput method to screen for NAT inhibitors.

### Structure-function comparisons of primate NAT proteins

The comparative approach undertaken in this study places the phylogeny of primate NAT homologues in the context of enzymatic function. The evolution of NAT enzymes has presumably been influenced by xenobiotic challenges conferred by the various habitats of primate species, particularly their diet. Although primate NAT proteins are highly conserved in amino acid sequence, they differ considerably in enzymatic activity (Fig. 2). Associating genetic variability with enzymatic function allows the identification of amino acid residues that may have been influenced by environmental factors during primate NAT evolution. Such comparisons may also reveal apparently subtle differences between NAT homologues that may still have a significant phenotypic impact, further advancing our knowledge of NAT catalytic mechanism. This approach is focusing only on functional variability developed through the slow action of natural selection, so it is complementary to artificial mutagenesis of recombinant proteins which usually aims to alter specific residues with presupposed functional consequences^31,57–59^. To this end, some interesting comparisons are discussed below.

#### (*MACMU/MACSY*)*NAT1 vs*. (*MANSP*)*NAT1*

As mentioned above, the macaque (MACMU/MACSY)NAT1 was demonstrated to be considerably more active compared with other primate NAT1 homologues (Fig. 2a). It shares highest phylogenetic and primary sequence similarity (98.63%) with (MANSP)NAT1 which, however, provided 55-fold lower specific activity with the selective substrate PABA. Both proteins were very effective during recombinant expression/purification (Fig. 1) and (MANSP)NAT1 had a higher Tm by 3.4 °C (Fig. 5a and c). Therefore, inherent instability of (MANSP)NAT1 protein is unlikely to explain its lower enzymatic activity relative to (MACMU/MACSY)NAT1. At the primary sequence level, the two homologues vary only at amino acid positions 29, 95, 273 and 280 (Supplementary Fig. S2). (MACMU/MACSY)NAT1 was the only NAT1 homologue in the study with glutamate at position 29, instead of glutamine. It was also the only NAT1 homologue with asparagine, instead of serine, at position 95. Conversely, (MANSP)NAT1 was the only homologue with valine, instead of isoleucine, at position 273. Position 280 is less conserved among the compared NAT1 sequences, bearing a methionine in (MACMU/MACSY)NAT1, an isoleucine in (MANSP)NAT1 and a valine or methionine in other primates.

**Figure 7:**
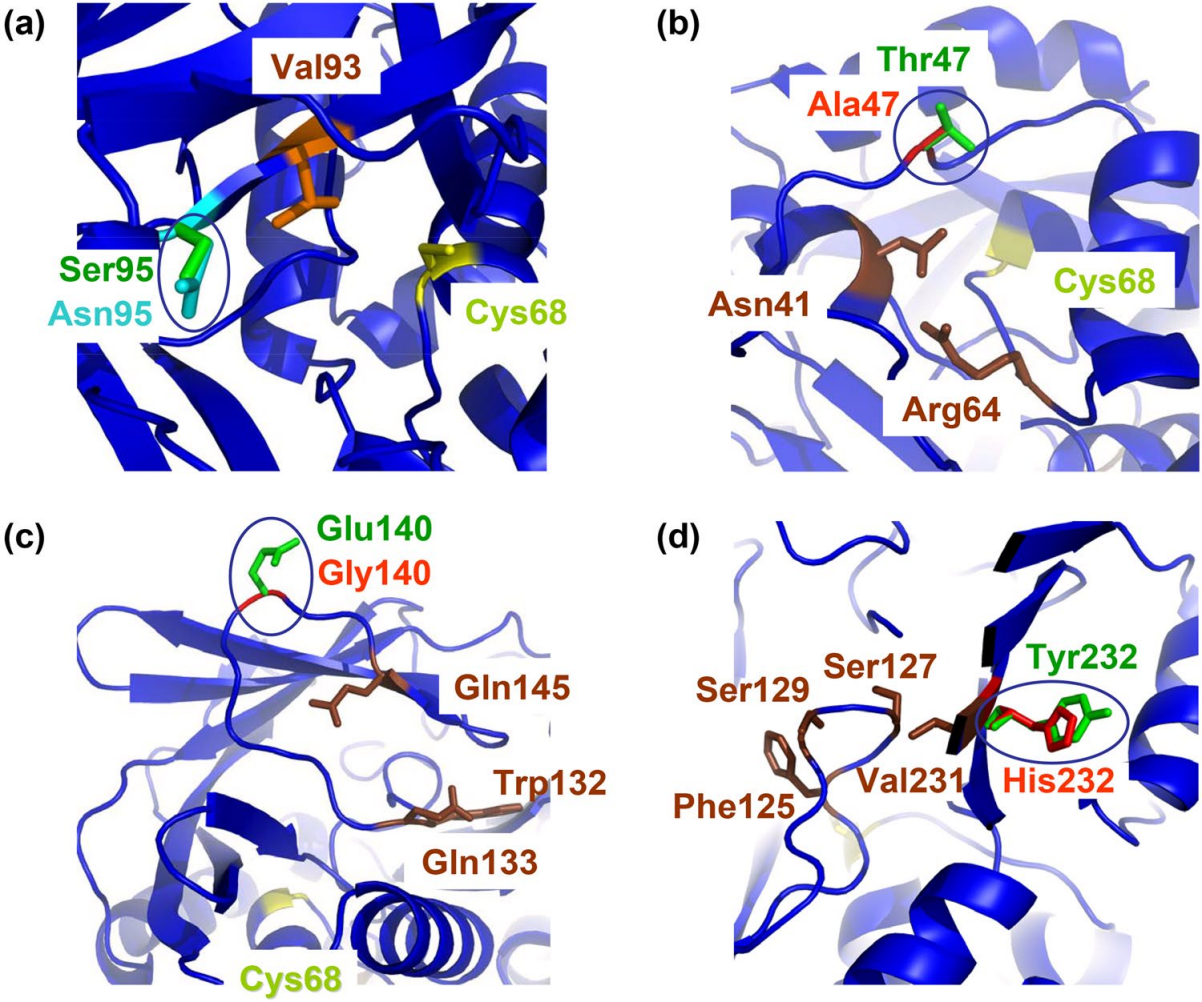
Examples of amino acid residues potentially differentiating the enzymatic function of highly homologous NAT proteins of non-human primates. (**a**) Detailed partial view of (MACMU/MACSY)NAT1, showing residue 95 (circled) and other proximal amino acids of relevance. Residue 95 is predicted to functionally differentiate (MACMU/MACSY)NAT1 (Asn95) from its 98.63% homologous (MANSP)NAT1 (Ser95). (**b**,**c**) Detailed partial view of (ERYPA)NAT2, showing circled residues 47 (**b**) and 140 (**c**), together with other proximal amino acids of relevance. Residues 47 and 140 are predicted to functionally differentiate (ERYPA)NAT2 (Ala47, Gly140) from its 99.31% homologous (CHLTN)NAT2 (Thr47, Glu140). (**d**) Detailed partial view of (MACMU)NAT2, showing residue 232 (circled) and other proximal amino acids of relevance. Residue 232 is predicted to functionally differentiate (MACMU)NAT2 (His232) from its 99.31% homologous (MACSY)NAT2 (Tyr232). The NAT1 and NAT2 homologues of non-human primates were modelled against the crystal structure of (HUMAN)NAT1 (PDB ID: 2PQT) and (HUMAN)NAT2 (PDB ID: 2PFR), respectively.

Variant residue 29 is located at the α2-helix, on the surface of the protein molecule and away from the active site. In contrast, residue 95 is positioned within the catalytic pocket, next to the highly conserved Val93 which has been identified as one of the key hydrophobic residues involved in binding of NAT1 selective substrates^23^ (Fig. 7a). More sophisticated structure-function investigations may reveal if the bulkier Asn95 of (MACMU/MACSY)NAT1 might confer increased enzymatic activity, relative to (MANSP)NAT1 and other primate homologues with serine at this position. Residues 273 and 280, on the other hand, are unlikely to directly interact with the active site, although they are located at the beginning of the carboxy-terminal tail, which in human NATs is folding into the catalytic pocket where it interacts with CoA^23^.

#### (*ERYPA*)*NAT2 vs*. (*CERDI*)*NAT2 and* (*CHLTN*)*NAT2*

Among the primate NAT2 homologues examined, (ERYPA)NAT2 combined highest enzymatic activity with increased thermal stability (Figs 2b and 5b,d). It shares 99.66% and 99.31% primary sequence identity with its phylogenetically closest homologues (CERDI)NAT2 and (CHLTN)NAT2, respectively. Compared with other NAT2 homologues, those three proteins demonstrated robust recombinant expression and purification (Fig. 1). (ERYPA)NAT2 and (CERDI)NAT2 produced comparable levels of enzymatic activity which were considerably higher (10- to 20-fold with PANS) relative to (CHLTN)NAT2 (Fig. 2b). The latter protein also had the lowest Tm of the three, suggesting decreased stability (Fig. 5b and d).

(ERYPA)NAT2 differs from (CERDI)NAT2 only at amino acid position 276. Unlike other NAT2 sequences in this study, which have a glycine at position 276, (CERDI)NAT2 has a glutamate (Supplementary Fig. S2). This residue is located in the carboxy-terminal tail of NAT2, on the surface of the protein molecule, and has no apparent interaction with other amino acids. This single difference between (ERYPA)NAT2 and (CERDI)NAT2 is, therefore, unlikely to have any significant consequence on enzymatic function, consistent with the experimental findings.

(CHLTN)NAT2 is different from (ERYPA)NAT2 by two amino acids at positions 47 and 140. (CHLTN)NAT2 has a threonine and a glutamate, unlike other investigated NAT2 homologues which have an alanine and a glycine at these positions, respectively (Supplementary Fig. S2). The residue at position 47 is located behind the α-helix of the catalytic cysteine, and is also within spatial proximity to Asn41 and Arg64 which interact via hydrogen bonding to stabilise the active site^23^. It is possible that the bulkier polar threonine of (CHLTN)NAT2 destabilises this interaction, as opposed to the smaller hydrophobic alanine of (ERYPA)NAT2 and other NAT2 homologues (Fig. 7b). On the opposite side of the catalytic core, residue 140 is located right at the turn of an important loop stabilised through hydrogen bonding between Trp132, Gln133 and Gln145^23^. It is possible that the bulky negatively charged Glu140 of (CHLTN)NAT2 decreases the stability of this loop, in contrast to the small glycine of (ERYPA)NAT2 and other NAT2 homologues (Fig. 7c).

Polymorphisms affecting amino acid residues 64 (NAT2*14 and NAT2*19 alleles) and 145 (NAT2*17 allele) have been reported for human NAT2 and they confer the slow acetylator phenotype (http://nat.mbg.duth.gr/). Slow acetylator variants are very common in human populations^16^ and this should be expected to be the norm for populations of other primates too. It is, therefore, possible that the low activity of certain NAT homologues in this study is due to slow acetylator variants present in the single individual sampled per each primate species. To date, only two NAT polymorphic variants have been reported in the literature for (MACMU)NAT2 and these are functionally differentiated by a valine to isoleucine substitution at position 231^60,61^. Genotyping of a larger number of individuals would allow insight into *NAT* gene polymorphism in primates other than human.

#### (*MACSY*)*NAT2 vs*. (*MACMU*)*NAT2*

Another interesting observation arises from comparison of (MACSY)NAT2 and (MACMU)NAT2 belonging to the two macaque species (genus *Macaca*). As discussed above, the two homologues have 99.31% amino acid sequence identity, but the enzymatic activity of (MACSY)NAT2 was up to 3-fold higher relative to (MACMU)NAT2 (Fig. 2b). Among all investigated NAT2 proteins, (MACSY)NAT2 provided highest and (MACMU)NAT2 lowest Tm values which were different by about 6 °C (Fig. 5b and d). Moreover, recombinant expression of (MACSY)NAT2 was considerably more efficient compared with (MACMU)NAT2 (Fig. 1). The two homologues are different by only two amino acids at positions 155 and 232, where (MACSY)NAT2 has glutamate and tyrosine, and (MACMU)NAT2 has glutamine and histidine, respectively (Supplementary Fig. S2).

Among all primate NAT proteins investigated, glutamine is found at position 155 only in (MACMU)NAT2 (Supplementary Fig. S2). However, this residue is located on the surface of the protein molecule and so it is predicted to have limited impact. Position 232, on the other hand, is located in the middle of β13-sheet, one of the four anti-parallel β-sheets of domain III. Sheets β12–β15 form a wall just behind loop 125–129 comprising important amino acids that determine substrate selectivity of human NAT isoenzymes^23,31^. The intermediate space is also accessible to the tip of the carboxy-terminal tail. Residue 232 is located right next to the above-mentioned (MACMU)NAT2 polymorphic site p.Val231Ile, which sterically interacts with loop 125–129^61^. Although the side chain of residue 232 is oriented to the opposite direction from loop 125–129, it is possible that the size and polarity of amino acid 232 may influence the structural integrity of β13-sheet, with impact on loop 125–129 and the carboxy-terminal chain (Fig. 7d).

## Conclusions

This study undertook comparative functional investigation of NAT homologues from 10 non-human primates, in order to characterise their properties and evaluate their suitability as models of human NATs, particularly for *in vitro* screens requiring large quantities of pure recombinant protein. Considering factors, such as the overall amount of expressed recombinant protein, the enzymatic activity, the thermal stability and the percent identity to human NATs, certain homologues are proposed as more appropriate, i.e. (MACMU/MACSY)NAT1 and (ERYPA)NAT2, as well as the two NAT homologues of the gibbon *N*. *gabriellae* which show higher homology to human NATs. The practicality of using such homologues (instead of hard-to-express human NAT proteins) for standard *in vitro* screens lies in that the NAT proteins of non-human primates demonstrate higher sequence identity to human NATs (compared with previously used rodent homologues), and in that recombinant protein production is readily achievable once the *NAT* genes of non-human primates have been amplified and cloned from tiny amounts of genomic DNA. However, currently available rodent models for NAT^20^ should remain relevant, if suitable compounds become validated as candidates for *in vivo* preclinical tests in the future. Moreover, the comparative investigation of *NAT* genes and their enzyme products in non-human primates allows better understanding of their role in xenobiotic metabolism and facilitates the identification of functional residues that may have been subject to evolutionary pressures. To that end, genotyping of multiple individuals from different non-human primate species may help identify genetic variability similar to that observed for *NAT1* and *NAT2* genes in human populations.

## Methods

### Generation of gene constructs

Polymerase chain reaction (PCR) was performed with *Pfu*-DNA polymerase, using 10 ng of genomic DNA template. Primers (VBC Biotech) incorporating restriction sites at the ends of each PCR product were used according to the scheme of Table 1, and the primer annealing temperature was 56 °C. Subsequent restriction cloning into the pET28b(+) plasmid was carried out as described^61^. When necessary, site-directed mutagenesis was performed with the QuikChange II kit (Agilent) and appropriate mutagenic primers. The insert of each construct was sequenced (GATC Biotech) with vector-specific primers to verify in-frame translation into the expected N-terminal hexa-histidine tagged NAT amino acid sequence. Computational analyses of sequences (e.g. alignments, calculation of percent identities and phylogeny) were carried out with BioEdit Sequence Alignment Editor and MEGA^62^. All *NAT* gene constructs were maintained as clones in *E*. *coli* strain BL21(DE3)pLysS (Promega).

### Expression and chromatographic purification of recombinant proteins

Expression of recombinant proteins was performed in 250 ml of Terrific Broth medium with 50 μg/ml kanamycin, during overnight incubation at 15 °C (120 rpm), after induction with 1 mM isopropyl-1-thio-D-galactopyranoside (IPTG) at culture optical density (600 nm) of approximately 1. Soluble fractions of bacterial lysates were recovered in 20 mM Tris-HCl (pH 7.5), 500 mM NaCl, 0.1% (w/v) 3-[(3-cholamidopropyl)dimethylammonio]-1-propanesulfonate (CHAPS), 5 mM imidazole, 5% (v/v) glycerol, 2 mM β-mercaptoethanol and a commercial cocktail of protease inhibitors. Affinity chromatography took place through Ni-NTA resin (Qiagen) and recombinant proteins were eluted with increasing amounts of imidazole (25, 50, 100 and 250 mM). Procedures were performed as described^10^.

For proteins further purified by ion exchange and gel filtration chromatography, recombinant expression took place in 500 ml cultures and affinity chromatography followed according to the procedures above. Ion exchange chromatography was performed with an ÄKTA Purifier Core System carrying a positively charged Mono Q 5/50 GL column (GE Healthcare). This column was appropriate, as the isoelectric point (pI) of NAT proteins was calculated to range between 5 and 6, and the protein molecules were thus expected to be negatively charged in the chromatographic buffer used (pH 7.4). A concentration gradient (50 mM to 1 M) of NaCl was achieved by gradual mixing of buffer A (20 mM Tris-HCl pH 7.4, 50 mM ɛ-aminocaproic acid, 5 mM EDTA) with buffer B (buffer A plus 1 M NaCl), and the procedure was monitored by measuring absorbance at 280 nm.

Gel filtration chromatography was performed using a Superdex 75 10/300 GL column attached to the ÄKTA Purifier Core System (GE Healthcare). The expected molecular weight of mammalian NAT proteins is 30–35 kDa. Protein separation took place in buffer containing 20 mM Tris-HCl (pH 7.5), 300 mM NaCl, and the procedure was monitored at 280 nm. Molecular weight standards (Sigma-Aldrich) comprised proteins (0.5 mg each) bovine serum albumin (66.8 kDa), ovalbumin (44.3 kDa), soybean trypsin inhibitor (21.5 kDa) and bovine heart cytochrome c (12.4 kDa), which were eluted at volumes of 3.5, 4.5, 6 and 7 ml, respectively.

Buffer change of protein preparations was performed between different chromatographic procedures by overnight (4 °C) dialysis through 10 kDa molecular weight cut-off pore size membrane (Sigma-Aldrich). Concentration of protein solutions was carried out by centrifugation (4 °C) through filter devices (Ultracel YM-3, Microcon) with molecular weight cut-off pore size of 3 kDa. Removal of the N-terminal hexa-histidine tag of recombinant proteins was achieved via overnight (4 °C) cleavage by 1 U of human plasma thrombin (Sigma-Aldrich) per milligram of protein in 20 mM Tris-HCl (pH 7.5), 300 mM NaCl. Protein concentration was determined spectrophotometrically at 280 nm, and preparations were inspected by sodium dodecyl sulphate polyacrylamide gel electrophoresis (SDS-PAGE) after each purification process. Yields of incompletely purified recombinant proteins were estimated by densitometric analysis of gels, as described^61^.

### Enzyme activity and inhibition assays

The colorimetric method with Ellman’s reagent (5,5′-dithiobis-2-nitrobenzoic acid) was used to measure NAT enzyme activity of recombinant proteins^63^, following our standardised in-house protocol^10,61^. Replicate reactions were performed with acetyl-CoA (CID: 444493), *n*-propionyl-CoA (CID: 92753), malonyl-CoA (CID: 644066), succinyl-CoA (CID: 92133) or hexanoyl-CoA (CID: 449118) as donor substrates, and 2-aminophenol (2AP; CID: 5801), 4-chloroaniline (CLA; CID: 7812), *p*-anisidine (PANS; CID: 7732), 4-phenoxyaniline (POA; CID: 8764), *p*-aminobenzoate (PABA; CID: 4876), 5-aminosalicylate (5AS; CID: 4075), 3,4-dichloroaniline (3,4DCA; CID: 7257), procainamide (PA; CID: 4913), *p*-aminobenzoylglutamate (pABGlu; CID: 196473), isoniazid (INH; CID: 3767) or hydralazine (HDZ; CID: 3637) as acceptor substrates. The amount of dimethylsulphoxide (DMSO) per reaction was 0.5% (v/v). All compounds were from Sigma-Aldrich.

Inhibition assays with a previously characterised human NAT1 selective naphthoquinone inhibitor^46^ were carried out using 1 μg of affinity chromatography purified recombinant NAT1 protein mixed with 10 μM of inhibitor in 20 mM Tris-HCl (pH 7.5), 5% (v/v) DMSO, prior to addition of 0.5 mM of substrate (PANS or PABA). Reactions were initiated with 0.5 mM acetyl-CoA and stopped after 30 s for colorimetric measurement of released CoA. Control reactions with 5% (v/v) DMSO but no inhibitor were also performed.

### Differential scanning fluorimetry

Differential scanning fluorimetry (DSF) was performed exactly as described^10,61^ to determine the denaturation midpoint temperatures (T_m_) of different NAT proteins, as well as to examine the effects of compound binding.

### Molecular modelling of protein structures

The published crystal structures^23^ of human NAT1 (PDB ID: 2PQT) and NAT2 (PDB ID: 2PFR) proteins were used to model the secondary and tertiary structures of NAT homologues from other primates. Alignments of non-human NATs to the secondary structure of human NATs were performed with T-COFFEE Expresso (http://tcoffee.crg.cat/apps/tcoffee/do:expresso)^64^ and graphically visualised with ESPript3.0 (http://espript.ibcp.fr)^65^. The tertiary structure of non-human NATs was modelled to the three-dimensional structure of human NATs using SWISS-MODEL (http://swissmodel.expasy.org/) and graphically visualised on PyMOL (Schrödinger, LLC). Docking analysis was performed as described^39^. In the available structure of human NAT1, the catalytic Cys68 appears modified as S-(2-anilino-2-oxoethyl)-L-cysteine and this modification was removed to restore the functional residue.

## Electronic supplementary material

Supplementary Information
