# Supplementary material for: Comparative analysis of xenobiotic metabolising N-acetyltransferases from ten non-human primates as in vitro models of human homologues
Source: Sci Rep. 2018 Jun 27;8:9759. doi: 10.1038/s41598-018-28094-6 (PMC6021393; doi:10.1038/s41598-018-28094-6)
Supplement: Supplementary file 1 — Supplementary Information [file 41598_2018_28094_MOESM1_ESM.pdf]

Supplementary Information

To manuscript entitled:

Comparative analysis of xenobiotic metabolising *N*-acetyltransferases from ten non-human primates as *in vitro* models of human homologues

By authors:

Theodora Tsirka, Maria Konstantopoulou, Audrey Sabbagh, Brigitte Crouau-Roy, Ali Ryan, Edith Sim, Sotiria Boukouvala, Giannoulis Fakis

(Supplementary components are provided in order of appearance in the manuscript)

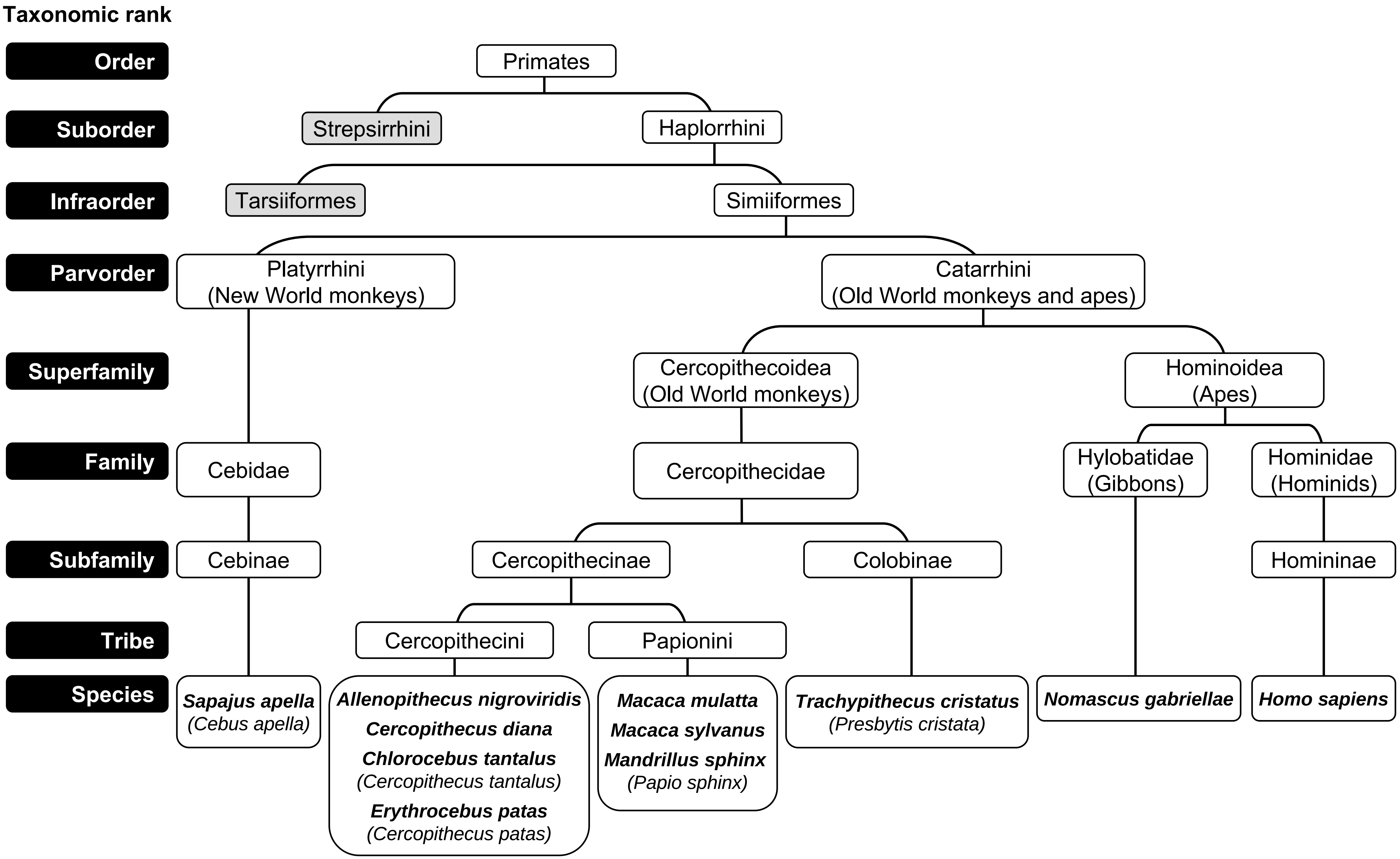

Supplementary Figure S1: Consensus taxonomic classification of compared primates. Eleven species of simians (infraorder of Simiiformes) are shown, with synonymous names in parentheses (if applicable). The information was retrieved from the NCBI Taxonomy Database (https://www.ncbi.nlm.nih.gov/taxonomy/) using the common tree display tool.

Supplementary Table S1: Geographic distribution and nutritional habits of compared primate species, classified as New World monkeys, Old World monkeys or apes (including human). The information was compiled from the Animal Diversity Web of the University of Michigan Museum of Zoology in the USA (http://animaldiversity.org/accounts/Primates/classification/#Primates).

**Species**	**Taxonomic group**	**Geographic range**	**Diet**
Allenopithecus nigroviridis	Old World monkeys	Central Africa	Omnivore (insects, leaves, fruit)
Cercopithecus diana	Old World monkeys	West Africa	Omnivore (insects, terrestrial non-insect arthropods, leaves, fruit, flowers)
Chlorocebus tantalus (Cercopithecus tantalus)	Old World monkeys	Central Africa	Omnivore (leaves, gum, seeds, nuts, grasses, fungi, fruit, berries, flowers, buds, shoots, invertebrates, eggs, birds, lizards, rodents and other vertebrate prey)
Erythrocebus patas (Cercopithecus patas)	Old World monkeys	Central Africa	Omnivore (eggs, insects, terrestrial non-insect arthropods, leaves, roots and tubers, fruit, flowers)
Homo sapiens	Apes	Global	Omnivore
Macaca mulatta	Old World monkeys	South Asia	Omnivore (birds, mammals, amphibians, reptiles, insects, leaves, roots and tubers, fruit)
Macaca sylvanus	Old World monkeys	Morocco, Algeria, Gibraltar	Omnivore (amphibians, insects, leaves, roots and tubers, wood, bark or stems, seeds, grains and nuts, fruit, flowers, fungus)
Mandrillus sphinx (Papio sphinx)	Old World monkeys	Central Africa	Omnivore (amphibians, reptiles, insects, mollusks, terrestrial worms, roots and tubers, seeds, grains and nuts, fruit, fungus)
Nomascus gabriellae	Apes	Southeast Asia	Mainly herbivore (leaves, fruit, flowers, insects)
Sapajus apella (Cebus apella)	New World monkeys	South America	Omnivore (birds, mammals, amphibians, reptiles, eggs, insects, terrestrial non-insect arthropods, leaves, roots and tubers, wood, bark or stems, seeds, grains and nuts, fruit, flowers)
Trachypithecus cristatus (Presbytis cristata)	Old World monkeys	Southeast Asia	Herbivore (leaves, seeds, grains and nuts, fruit, flowers)

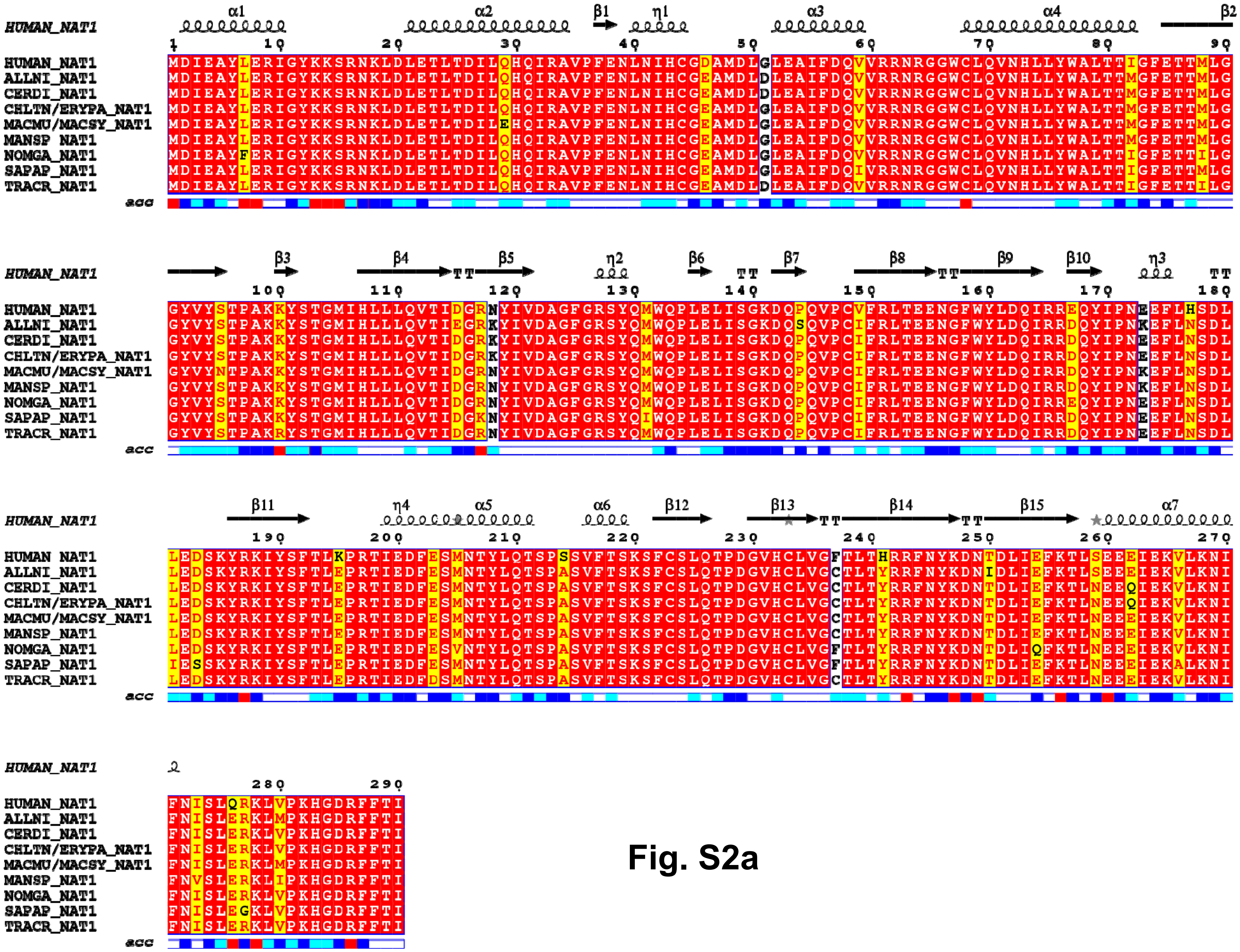
HUMAN_NAT1
α1 α2 β1 η1 α3 α4 β2
β3 β4 β5 η2 β6 β7 β8 β9 β10 η3
β11 η4 α5 α6 β12 β13 β14 β15 α7
HUMAN_NAT1
ALLNI_NAT1
CERDI_NAT1
CHLTN/ERYPA_NAT1
MACMU/MACSY_NAT1
MANSP_NAT1
NOMGA_NAT1
SAPAP_NAT1
TRACR_NAT1
acc

Fig. S2a

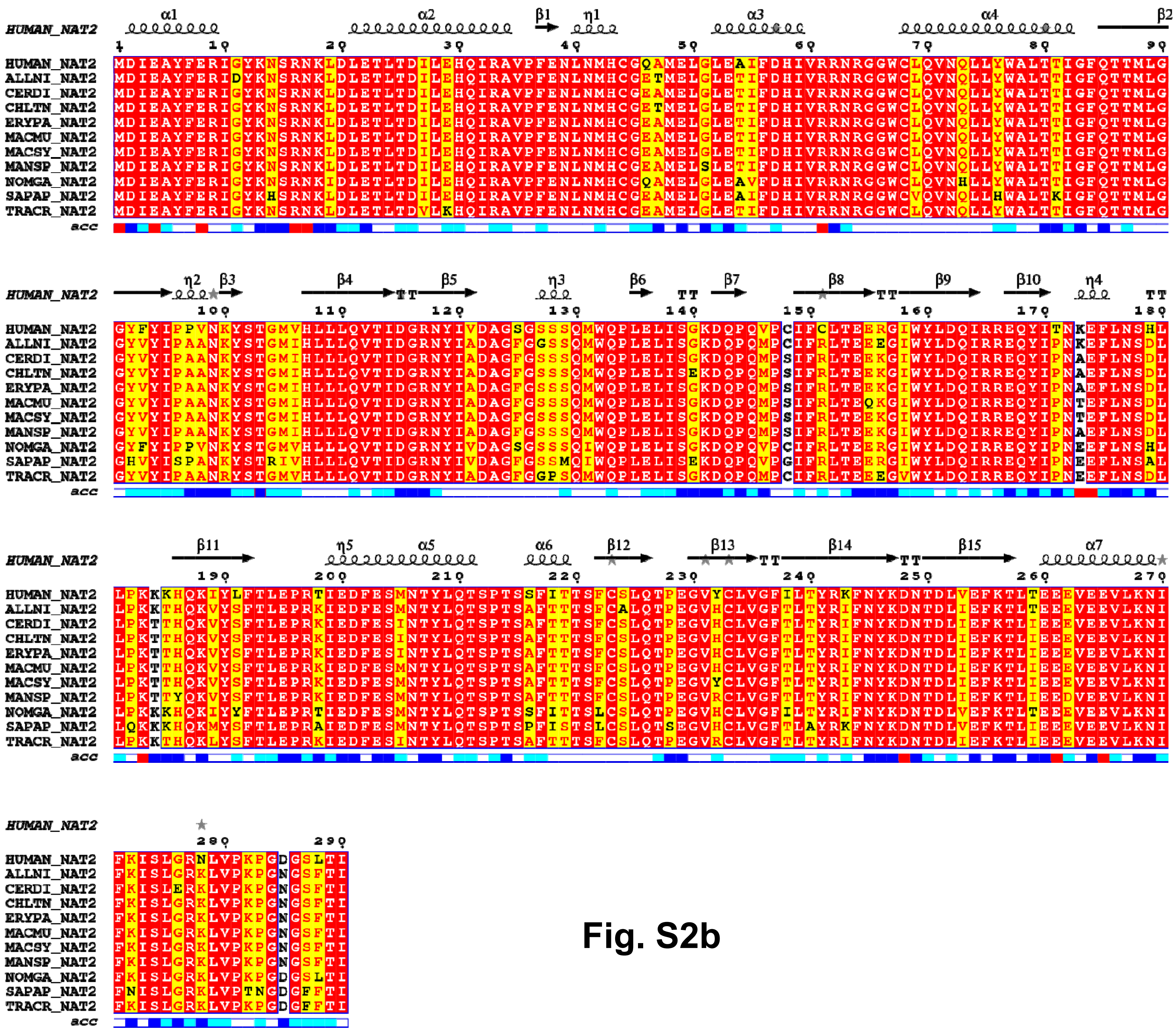
HUMAN_NAT2
α1 α2 β1 η1 α3 α4 β2
η2 β3 β4 β5 η3 β6 β7 β8 β9 β10 η4
β11 η5 α5 α6 β12 β13 β14 β15 α7
HUMAN_NAT2
ALLNI_NAT2
CERDI_NAT2
CHLTN_NAT2
ERYPA_NAT2
MACMU_NAT2
MACSY_NAT2
MANSP_NAT2
NOMGA_NAT2
SAPAP_NAT2
TRACR_NAT2
acc
1 10 20 30 40 50 60 70 80 90
100 110 120 130 140 150 160 170 180
190 200 210 220 230 240 250 260 270
280 290

Fig. S2b

Supplementary Figure S2: Structural alignments of NAT protein sequences from compared primate species.

The deduced amino acid sequences of NAT1 (Fig. S2a) and NAT2 (Fig. S2b) proteins from *Homo sapiens* (HUMAN), *Allenopithecus nigroviridis* (ALLNI), *Cercopithecus diana* (CERDI), *Chlorocebus tantalus* (CHLTN), *Erythrocebus patas* (ERYPA), *Macaca mulatta* (MACMU), *Macaca sylvanus* (MACSY), *Mandrillus sphinx* (MANSP), *Nomascus gabriellae* (NOMGA), *Sapajus apella* (SAPAP) and *Trachypithecus cristatus* (TRACR) were aligned to the secondary structural elements of human NAT1 (PDB ID: 2PQT) and NAT2 (PDB ID: 2PFR), respectively. It is noted that the NAT1 amino acid sequences were identical between *C. tantalus* and *E. patas*, as well as between *M. mulatta* and *M. sylvanus*, so the corresponding homologues are shown as (CHLTN/ERYPA)NAT1 and (MACMU/MACSY)NAT1, respectively. Below each alignment, residues are designated as accessible (blue), partly accessible (cyan) or buried (white), while red is not predicted.

Supplementary Table S2: Comparison of NAT protein sequences investigated.

The two parts show the percent identities of NAT1 (top) and NAT2 (bottom) amino acid sequences from *Homo sapiens* (HUMAN), *Allenopithecus nigroviridis* (ALLNI), *Cercopithecus diana* (CERDI), *Chlorocebus tantalus* (CHLTN), *Erythrocebus patas* (ERYPA), *Macaca mulatta* (MACMU), *Macaca sylvanus* (MACSY), *Mandrillus sphinx* (MANSP), *Nomascus gabriellae* (NOMGA), *Sapajus apella* (SAPAP) and *Trachypithecus cristatus* (TRACR). Comparison is also shown between the primate NAT1 sequences and the mouse NAT2 protein (top part), as well as between the primate NAT2 sequences and the mouse NAT1 protein (bottom part).

(HUMAN) NAT1	(ALLNI) NAT1	(CERDI) NAT1	(CHLTN) NAT1	(ERYPA) NAT1	(MACMU) NAT1	(MACSY) NAT1	(MANSP) NAT1	(NOMGA) NAT1	(SAPAP) NAT1	(TRACR) NAT1	(MOUSE) NAT2	
100	94.16	95.19	95.53	95.53	94.85	94.85	95.19	95.88	94.50	95.19	82.13	**(HUMAN)NAT1**
	100	97.59	97.25	97.25	97.25	97.25	97.25	94.85	94.16	96.22	83.51	**(ALLNI)NAT1**
		100	99.66	99.66	97.59	97.59	97.94	96.56	95.88	97.94	83.85	**(CERDI)NAT1**
			100	100	97.94	97.94	98.28	96.91	96.22	97.59	83.85	**(CHLTN)NAT1**
				100	97.94	97.94	98.28	96.91	96.22	97.59	83.85	**(ERYPA)NAT1**
					100	100	98.63	96.22	95.53	96.91	83.85	**(MACMU)NAT1**
						100	98.63	96.22	95.53	96.91	83.85	**(MACSY)NAT1**
							100	96.56	95.88	97.25	84.19	**(MANSP)NAT1**
								100	95.88	97.25	83.85	**(NOMGA)NAT1**
									100	95.88	83.51	**(SAPAP)NAT1**
										100	83.16	**(TRACR)NAT1**
											100	**(MOUSE)NAT2**

(HUMAN) NAT2	(ALLNI) NAT2	(CERDI) NAT2	(CHLTN) NAT2	(ERYPA) NAT2	(MACMU) NAT2	(MACSY) NAT2	(MANSP) NAT2	(NOMGA) NAT2	(SAPAP) NAT2	(TRACR) NAT2	(MOUSE) NAT1	
100	89.69	89.00	88.66	89.35	89.35	90.03	88.66	95.88	86.94	88.32	72.16	**(HUMAN)NAT2**
	100	96.22	96.56	96.56	96.56	96.56	95.53	89.35	85.57	94.50	71.82	**(ALLNI)NAT2**
		100	98.97	99.66	98.63	98.63	97.94	89.00	86.25	94.50	72.85	**(CERDI)NAT2**
			100	99.31	98.28	98.28	97.59	88.66	86.60	94.16	71.82	**(CHLTN)NAT2**
				100	98.97	98.97	98.28	89.35	86.60	94.85	72.51	**(ERYPA)NAT2**
					100	99.31	97.94	89.35	86.60	94.16	72.16	**(MACMU)NAT2**
						100	98.28	89.35	86.60	94.50	72.16	**(MACSY)NAT2**
							100	88.32	85.57	93.81	72.16	**(MANSP)NAT2**
								100	87.97	88.32	72.51	**(NOMGA)NAT2**
									100	85.91	72.16	**(SAPAP)NAT2**
										100	72.85	**(TRACR)NAT2**
											100	**(MOUSE)NAT1**

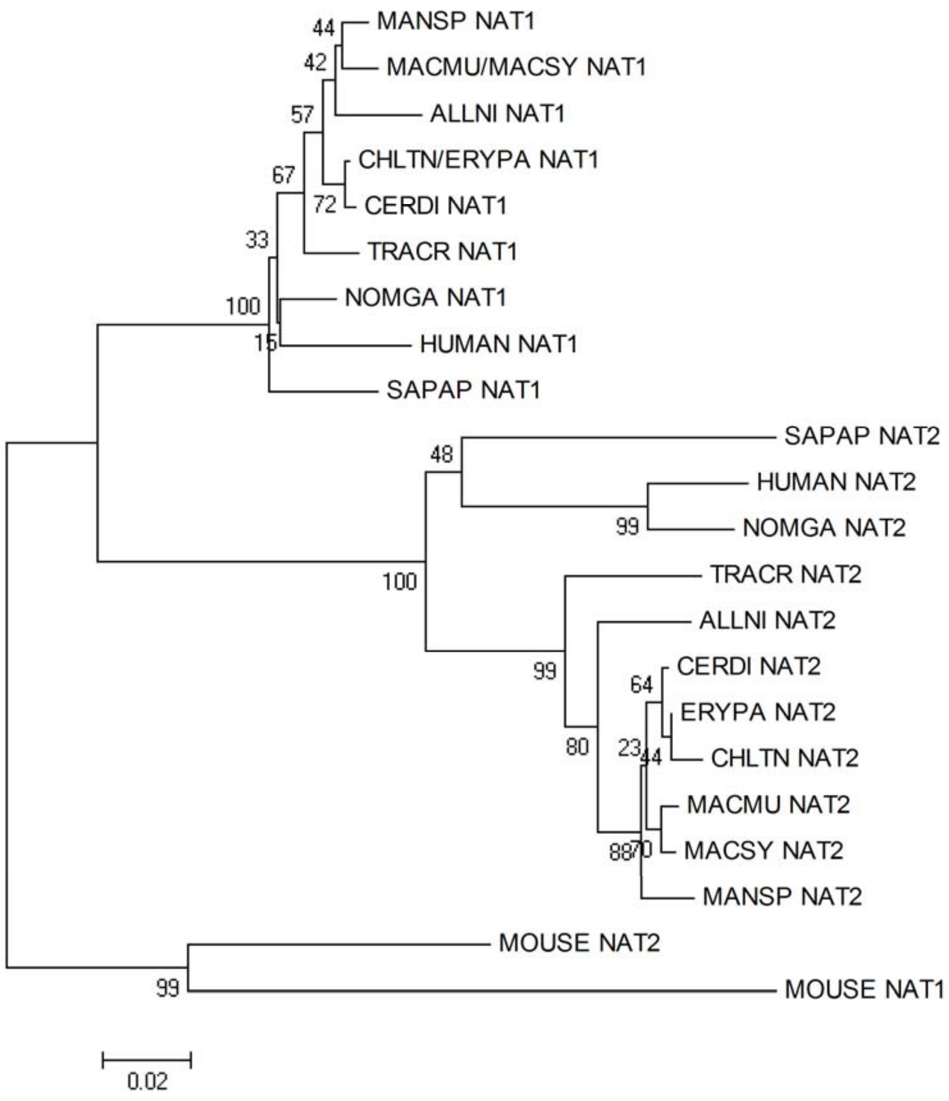

Supplementary Figure S3: Phylogenetic analysis of compared NAT sequences.

The deduced amino acid sequences of NAT1 and NAT2 homologues from *Allenopithecus nigroviridis* (ALLNI), *Cercopithecus diana* (CERDI), *Chlorocebus tantalus* (CHLTN), *Erythrocebus patas* (ERYPA), *Homo sapiens* (HUMAN), *Macaca mulatta* (MACMU), *Macaca sylvanus* (MACSY), *Mandrillus sphinx* (MANSP), *Nomascus gabriellae* (NOMGA), *Sapajus apella* (SAPAP) and *Trachypithecus cristatus* (TRACR) were used to construct the phylogenetic tree. The more divergent NAT1 and NAT2 protein sequences of *Mus musculus* (MOUSE) were used for comparison.

The Neighbour-Joining method was applied and the optimal tree with the sum of branch length =0.81734046 is shown. The percentage of replicate trees in which the associated sequences clustered together in the bootstrap test (2000 replicates) are shown next to the branches. The tree is drawn to scale, with branch lengths in the same units as those of the evolutionary distances used to infer the phylogenetic tree. The evolutionary distances were computed using the Poisson correction method and are in the units of the number of amino acid substitutions per site. There were a total of 290 positions in the final dataset with no gaps. Phylogenetic analysis was conducted in MEGA (Tamura et al (2007), *Molecular Biology and Evolution* 24:1596-1599).

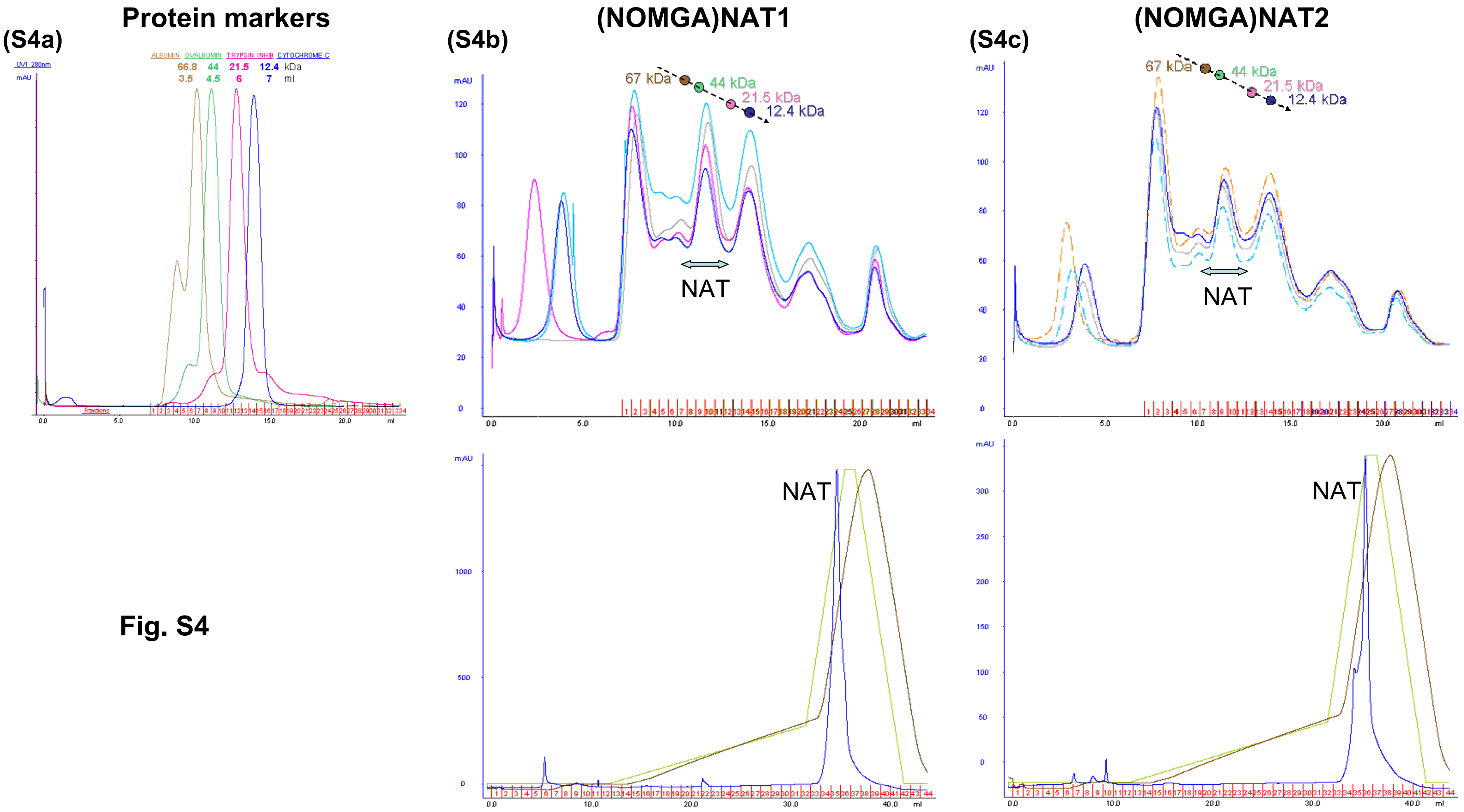
(S4a)
Protein markers
ALBUMIN OVALBUMIN TRYPSIN INHIB CYTOCHROME C
66.8 44 21.5 12.4 kDa
3.5 4.5 6 7 ml
UV1_280nm
mAU
Fractions
ml
(S4b)
(NOMGA)NAT1
67 kDa
44 kDa
21.5 kDa
12.4 kDa
NAT
mAU
ml
NAT
(S4c)
(NOMGA)NAT2
67 kDa
44 kDa
21.5 kDa
12.4 kDa
NAT
mAU
ml
NAT

Fig. S4

(S4d) (ALLNI)NAT1

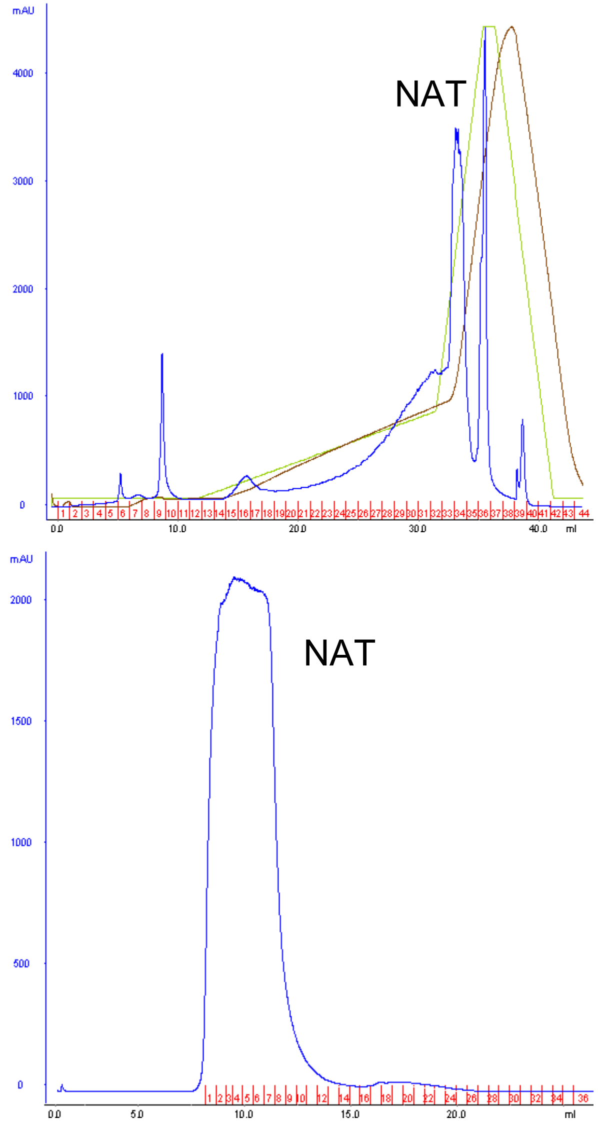

(S4e) (TRACR)NAT1

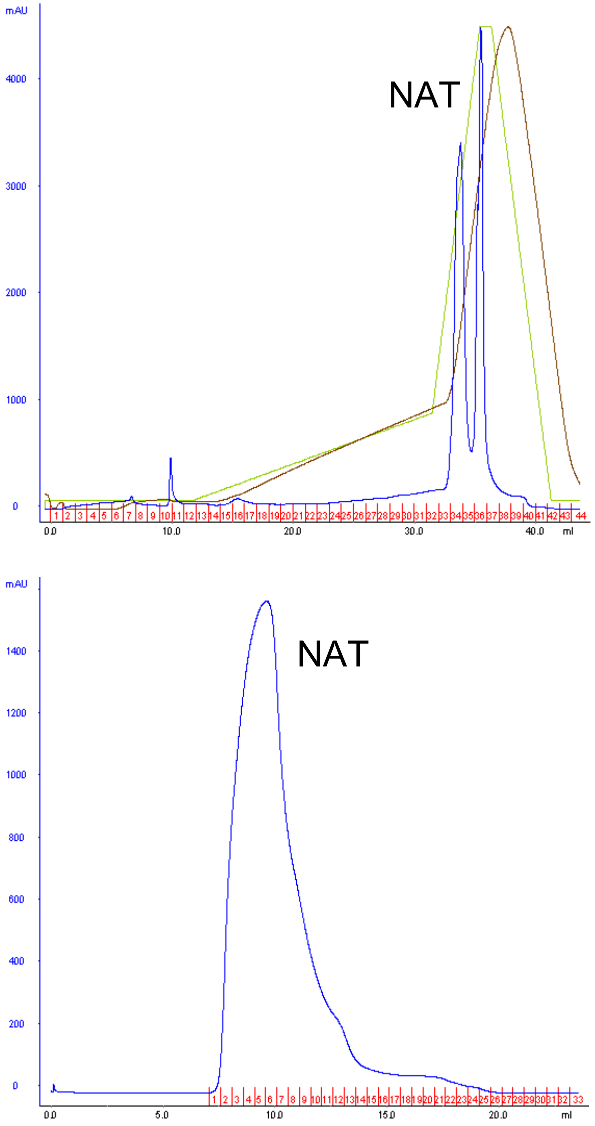

(S4f) (MANSP)NAT1

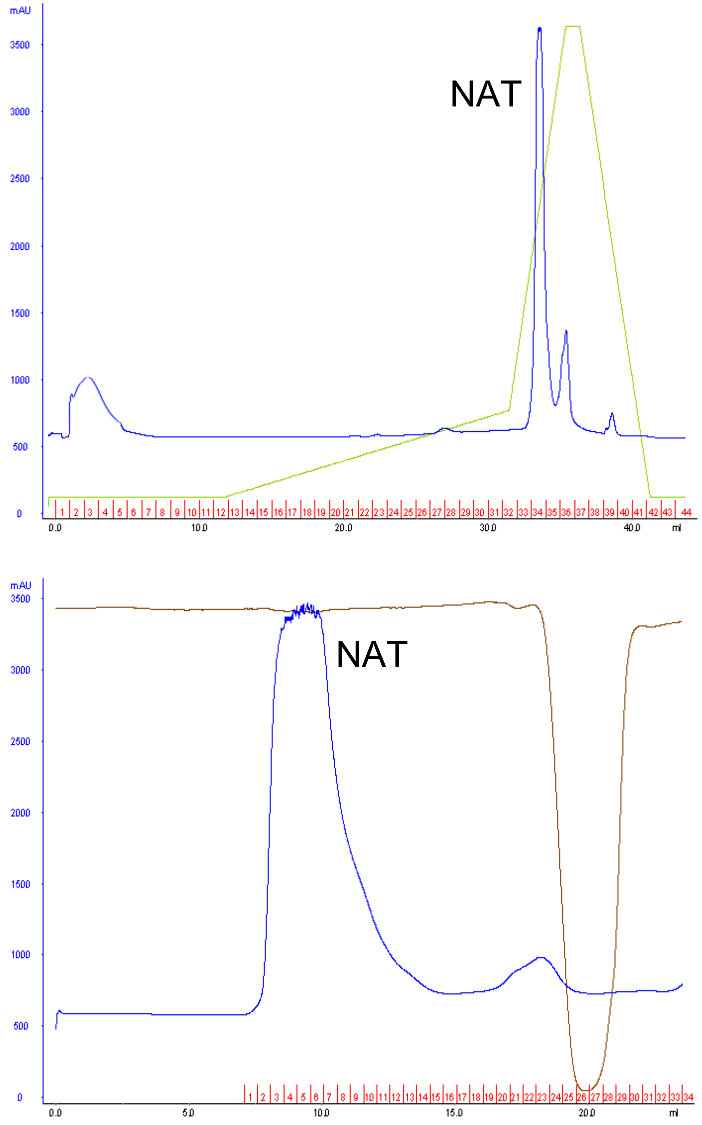

Supplementary Figure S4: Chromatographic purification of primate NAT recombinant proteins.

S4a: Gel filtration of four protein markers, shown as peaks coloured brown for bovine serum albumin (66.8 kDa, 3.5 ml elution volume), green for ovalbumin (44.3 kDa, 4.5 ml elution volume), pink for soybean trypsin inhibitor (21.5 kDa, 6 ml elution volume) and blue for bovine heart cytochrome c (12.4 kDa, 7 ml elution volume).

S4b-c: The (NOMGA)NAT proteins (~31 kDa) were eluted at volumes between 4.5 and 6 ml, as expected relative to the protein markers of Fig. S4a. The corresponding peaks are marked with a horizontal double arrow on gel filtration plots (top), which show successive runs of four aliquots of (NOMGA)NAT1 (Fig. S4b) and (NOMGA)NAT2 (Fig. S4c) preparations. The two proteins were then subjected to further purification by ion exchange chromatography (bottom).

S4d-f: Ion exchange chromatography (top), followed by gel filtration (bottom) of recombinant proteins (ALLNI)NAT1 (Fig. S4d), (TRACR)NAT1 (Fig. S4e) and (MANSP)NAT1 (Fig. S4f).

In all ion exchange chromatography plots, blue is the eluted protein (OD at 280 nm), green is the salt concentration and brown is the conductivity.

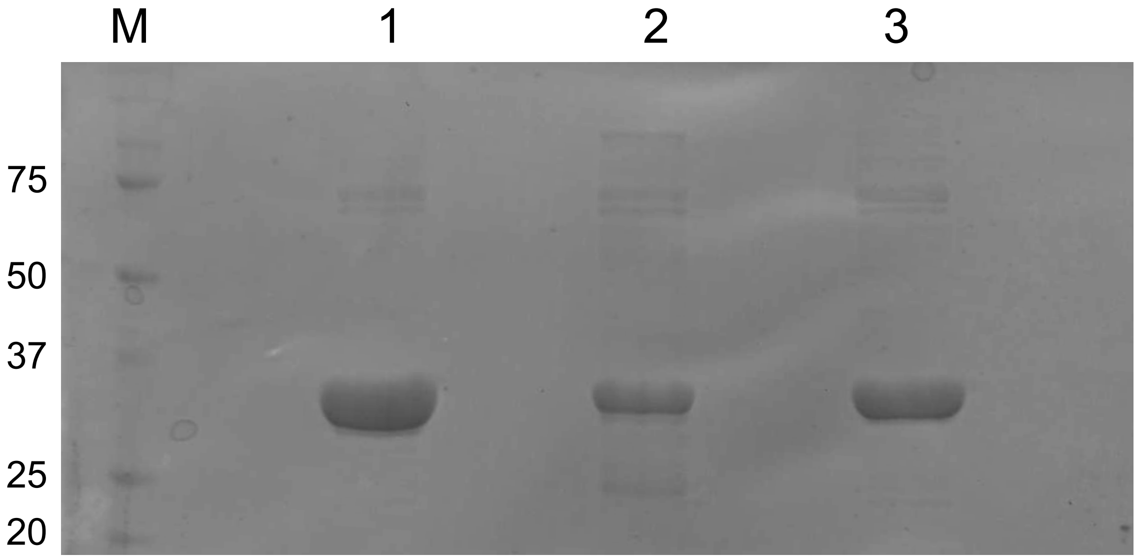

Supplementary Figure S5: Selected NAT proteins of non-human primates subjected to crystallography grade purification. The NAT1 proteins of *Allenopithecus nigroviridis* (ALLNI), *Nomascus gabriellae* (NOMGA) and *Trachypithecus cristatus* (TRACR) are shown in lanes 1-3, following successive chromatographic purifications (Supplementary Fig. S4). The preparations were finally concentrated to ~10 mg/ml, suitable for crystallographic screens. Lane M is Precision Plus Protein™ Standards (Bio-Rad), with bands of 20, 25, 37, 50 and 75 kDa marked on the left. The full-length gel is presented in Expanded Data Supplementary Figure 1 at the end of this Supplementary Information Section.

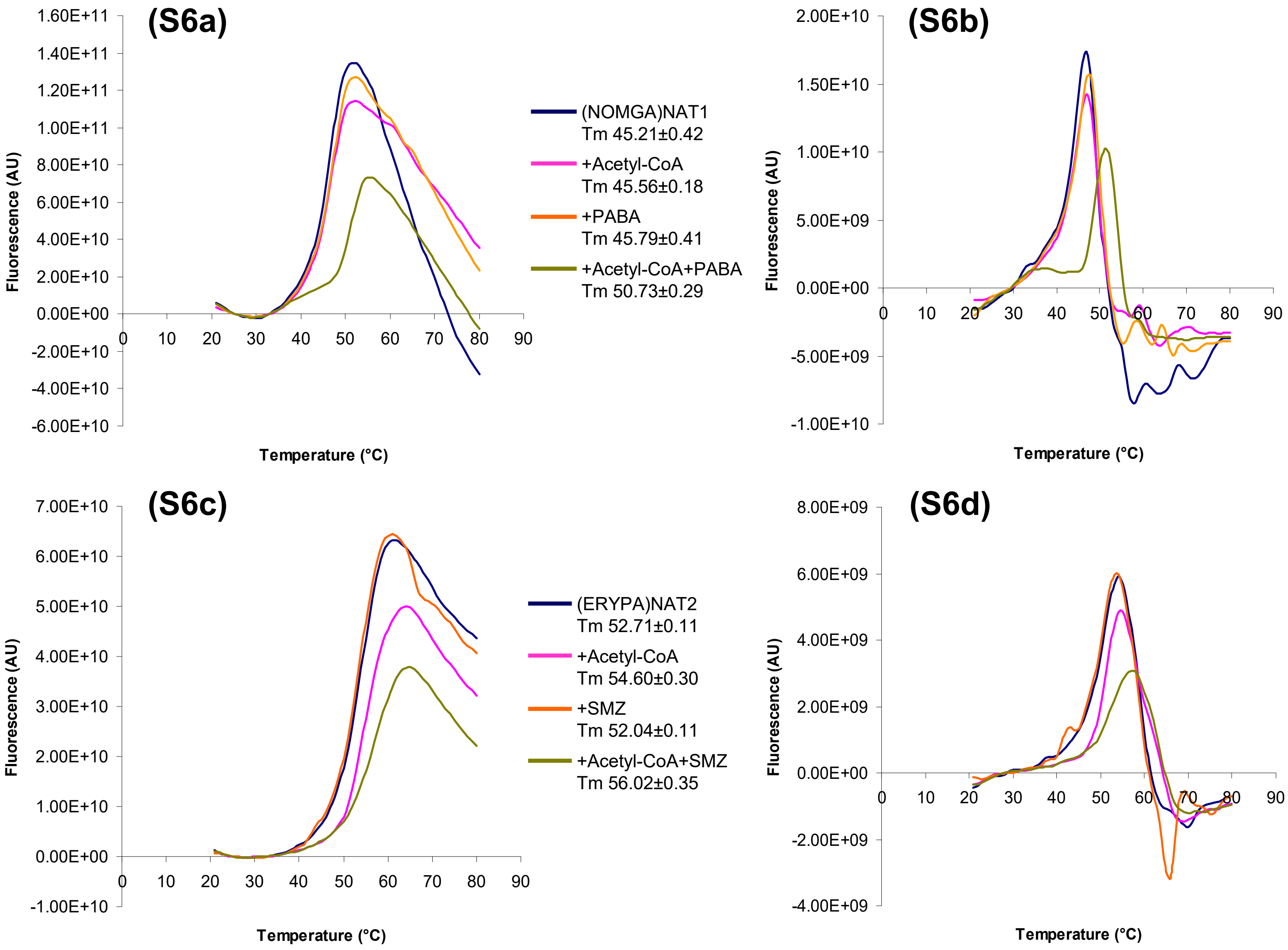

Supplementary Fig. S6: Example of primate NAT proteins assayed by differential scanning fluorimetry (DSF).

Thermal stability of recombinant NAT proteins was assessed with or without acetyl-CoA, arylamine or combinations of the two types of substrates. The panels on the left show the generated thermal denaturation curves, with change in SyproOrange fluorescence provided in arbitrary units (AU). The panels on the right show the derivative of generated thermal profiles, with main peaks indicating protein Tm values. Two replicate experiments were performed, producing overlapping curves for which the average plot is shown. The proteins used were (NOMGA)NAT1 of *Nomascus gabriellae* (Fig. S6a, S6b) and (ERYPA)NAT2 of *Erythrocebus patas* (Fig. S6c, S6d). The NAT1-selective *p*-aminobenzoate (PABA) and the NAT2-selective sulphamethazine (SMZ) substrates were used with or without acetyl-CoA, as described in the graph legends (Tm values provided in ^{o}C).

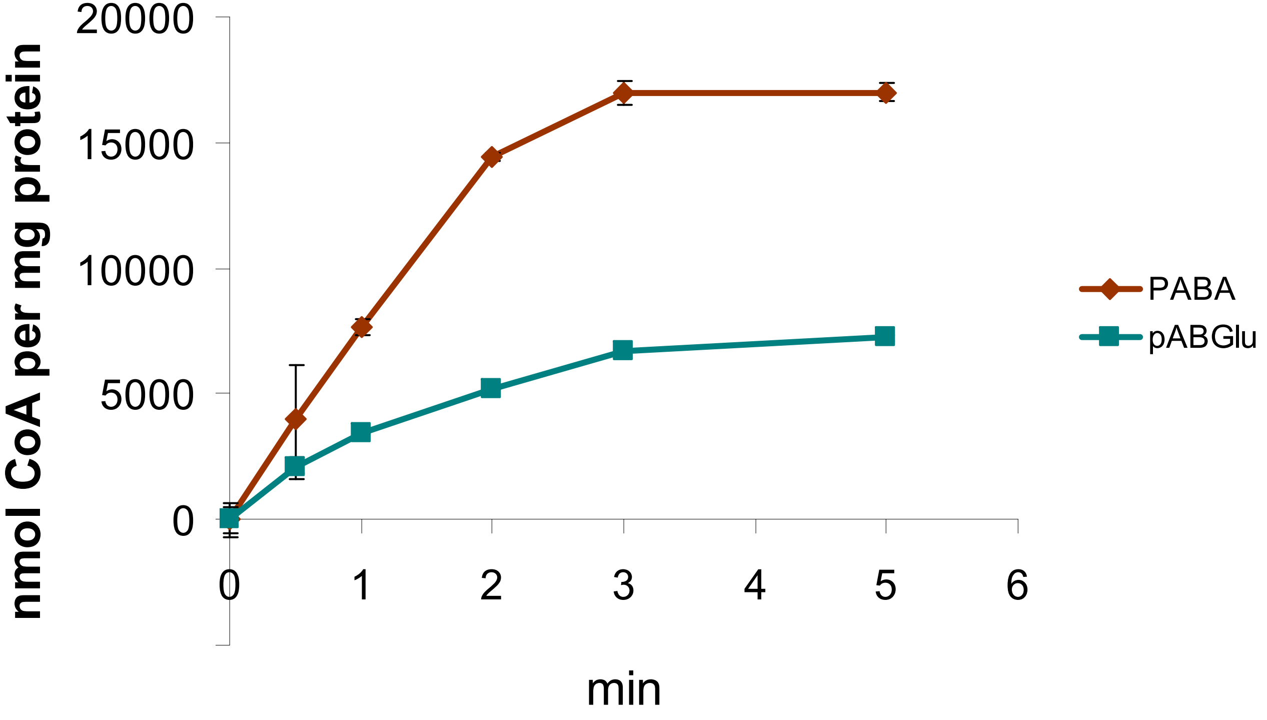

Supplementary Fig. S7: Primate NAT1 protein (non-human) assayed for enzymatic activity with PABA and pABGlu.

Enzymatic release of coenzyme A (CoA) was monitored with Ellman's reagent over specific time points, in reactions containing 1µg of purified (NOMGA)NAT1 protein from the gibbon *Nomascus gabriellae*, 0.4 mM of acetyl-CoA and 0.5 mM of either *p*-aminobenzoate (PABA) or *p*-aminobenzoylglutamate (pABGlu). The calculated enzymatic specific activity measured with PABA (7940 nmol/min/mg) was about 2-fold higher than with pABGlu (4104 nmol/min/mg). Duplicate reactions were performed and each data point is the average±standard deviation.

Expanded Data Supplementary Figures 1 & 2

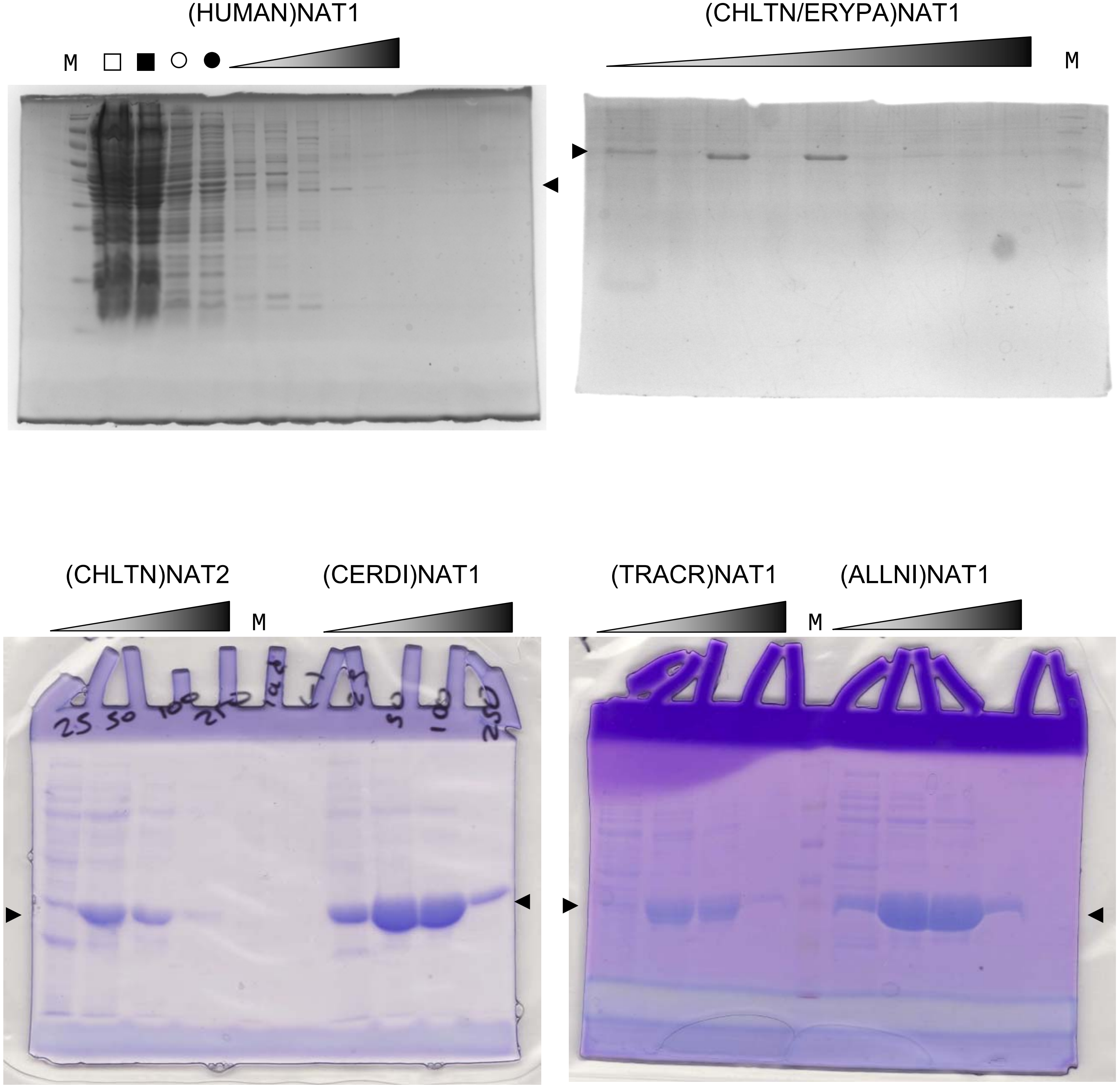
(HUMAN)NAT1
M
(CHLTN/ERYPA)NAT1
M
(CHLTN)NAT2
M
(CERDI)NAT1
(TRACR)NAT1
M
(ALLNI)NAT1

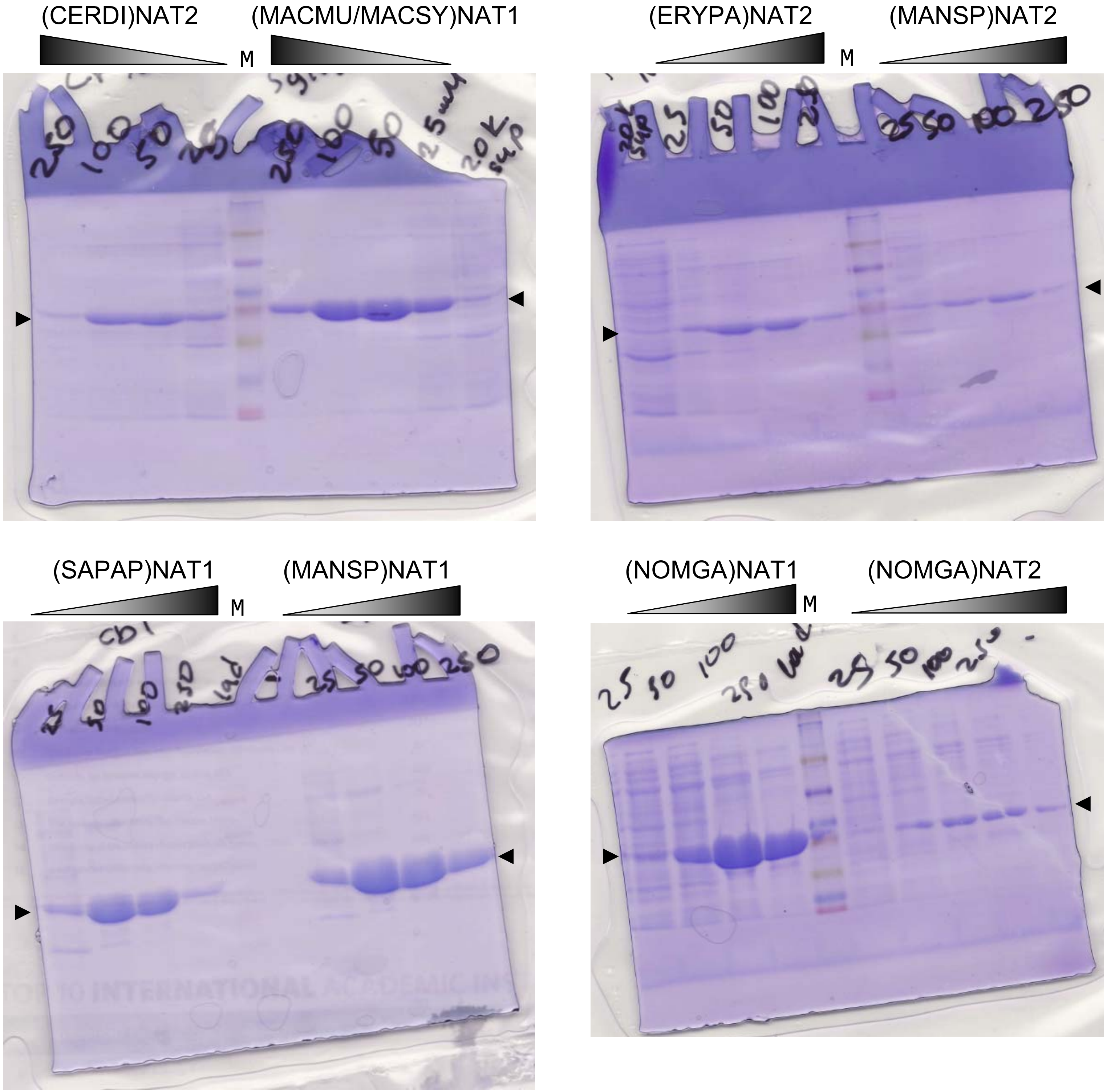
(CERDI)NAT2
(MACMU/MACSY)NAT1
M
(ERYPA)NAT2
M
(MANSP)NAT2
(SAPAP)NAT1
M
(MANSP)NAT1
(NOMGA)NAT1
M
(NOMGA)NAT2

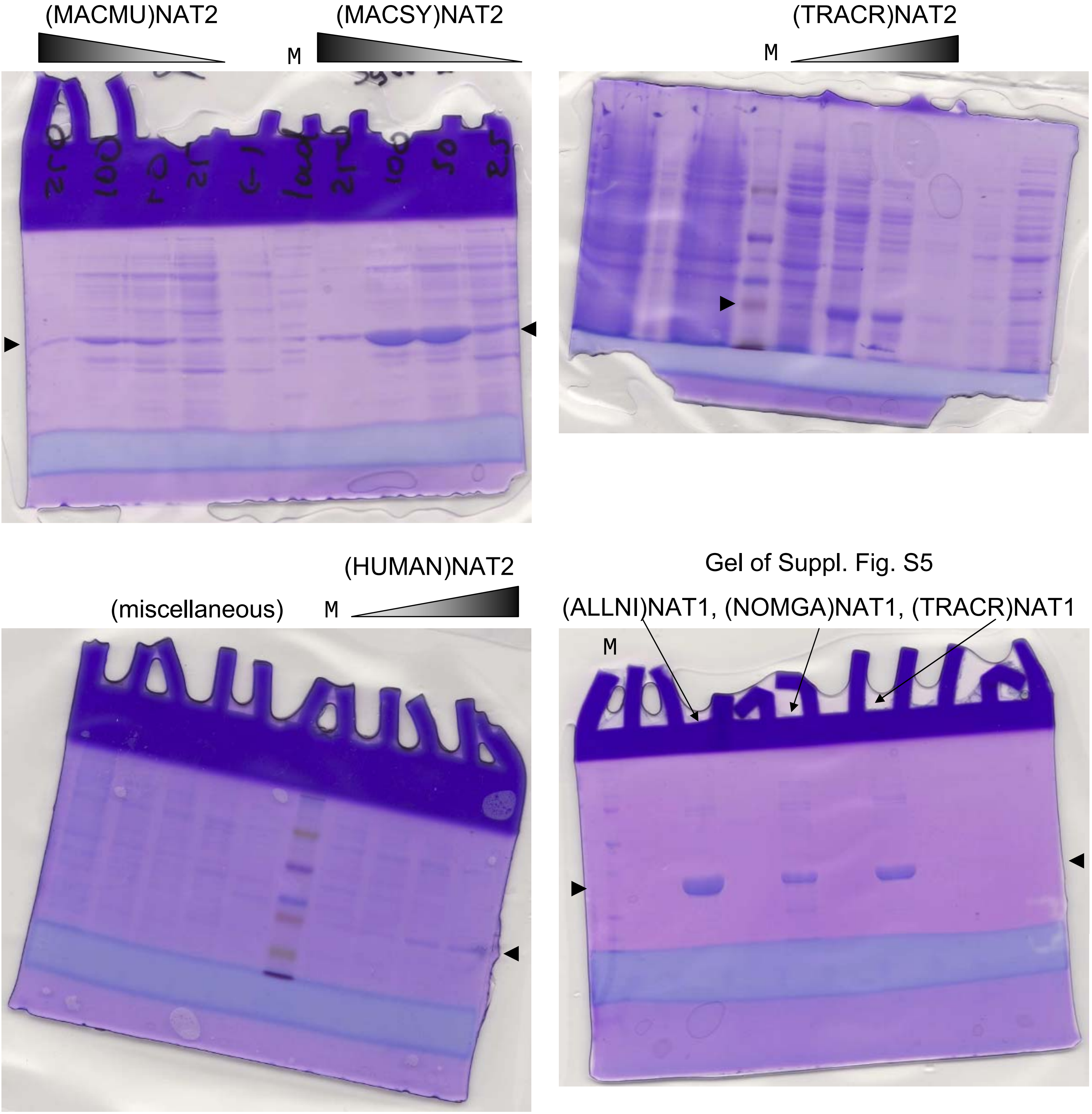
(MACMU)NAT2
(MACSY)NAT2
M
(TRACR)NAT2
M
(HUMAN)NAT2
(miscellaneous)
M
Gel of Suppl. Fig. S5
(ALLNI)NAT1, (NOMGA)NAT1, (TRACR)NAT1
M

Expanded Data Supplementary Figure 1: Full-length SDS-PAGE gels shown in the manuscript. The recombinant NAT proteins of Fig. 1 are presented, per gel, in the following order: (HUMAN)NAT1, (CHLTN/ERYPA)NAT1, (CHLTN)NAT2, (CERDI)NAT1, (TRACR)NAT1, (ALLNI)NAT1, (CERDI)NAT2, (MACMU/MACSY)NAT1, (ERYPA)NAT2, (MANSP)NAT2, (SAPAP)NAT1, (MANSP)NAT1, (NOMGA)NAT1, (NOMGA)NAT2, (MACMU)NAT2, (MACSY)NAT2, (TRACR)NAT2 and (HUMAN)NAT2. SDS-PAGE gels are shown with chromatographic fractions eluted with a concentration gradient of up to 250 mM of imidazole, indicated by shaded triangles at the top of each image. Each lane was loaded with 30 μl of eluate. The symbols in the first image are whole cell lysate without IPTG induction (□), whole cell lysate after IPTG induction (■), soluble cell extract before the affinity chromatography (○), and initial flowthrough of soluble cell extract through the affinity chromatography column (●). The small black arrowheads indicate bands of recombinant NAT proteins, the molecular weight of which (~31 kDa) was estimated relative to protein markers (lanes M).

All preparations were generated (over a period of two months) in the U.K. lab using identical conditions, and gels were scanned with a conventional office scanner. Exceptions are the first two gels shown, which represent our most successful attempts to generate (HUMAN)NAT1 and (CHLTN/ERYPA)NAT1 recombinant proteins under the standard conditions applied. These preparations were produced later in the Greek lab and the gels were photographed on a standard white-light transilluminator apparatus.

The final gel shows the fully purified recombinant proteins (ALLNI)NAT1, (NOMGA)NAT1 and (TRACR)NAT1 of Supplementary Fig. S5, generated and electrophoresed in the U.K. lab.

Panel 1

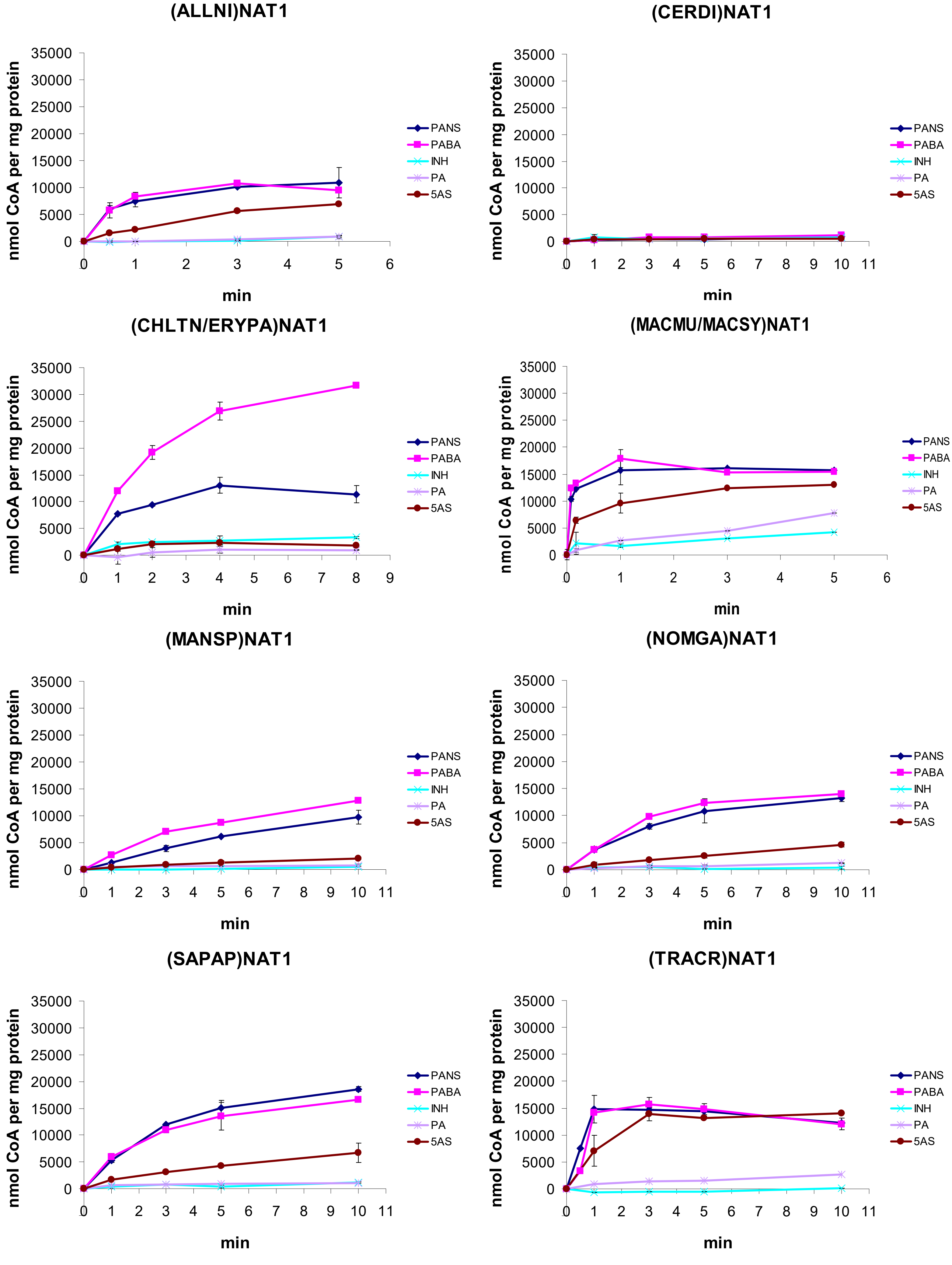
(ALLNI)NAT1
(CERDI)NAT1
(CHLTN/ERYPA)NAT1
(MACMU/MACSY)NAT1
(MANSP)NAT1
(NOMGA)NAT1
(SAPAP)NAT1
(TRACR)NAT1
nmol CoA per mg protein
min
PANS
PABA
INH
PA
5AS

Panel 2

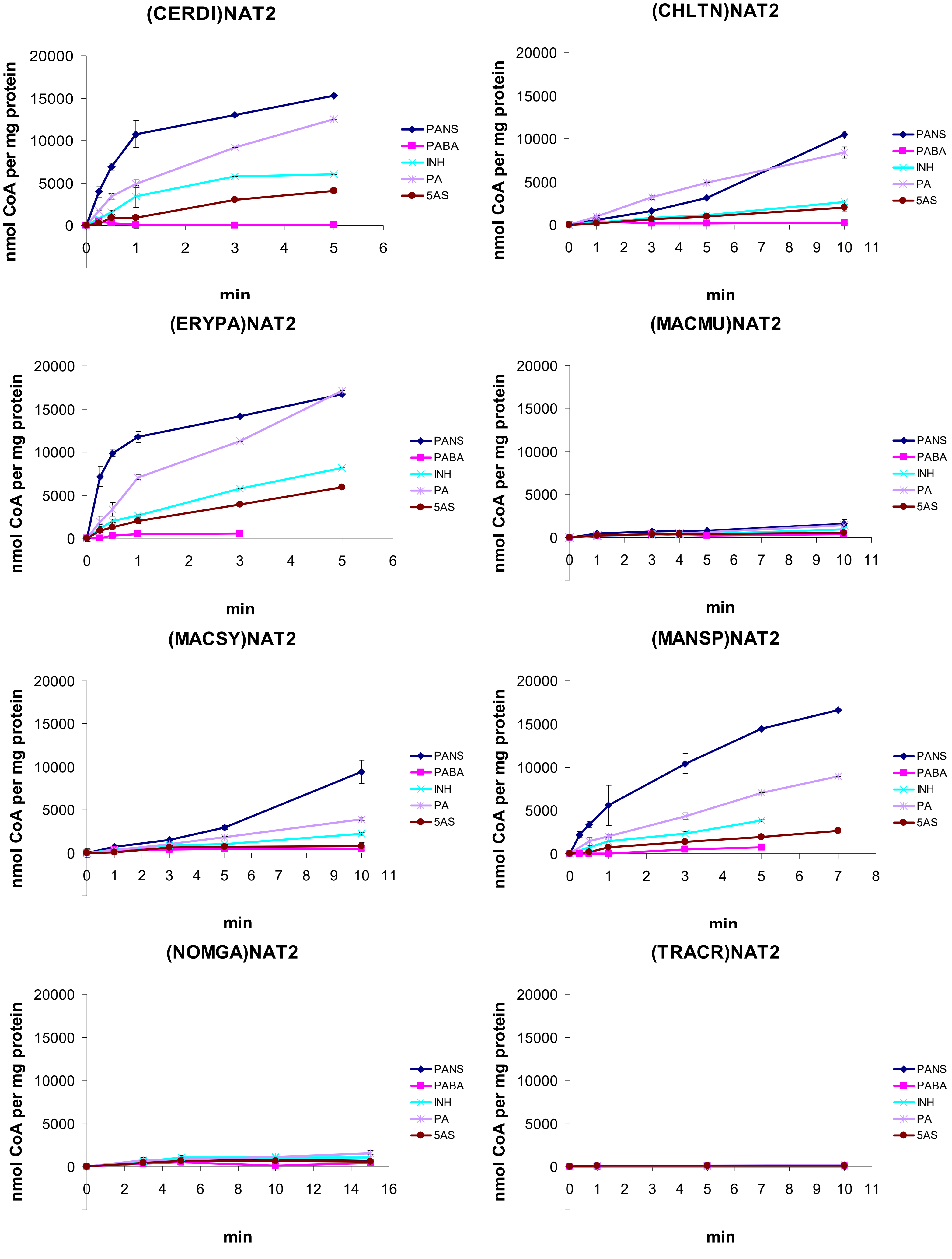

(CERDI)NAT2
(CHLTN)NAT2
(ERYPA)NAT2
(MACMU)NAT2
(MACSY)NAT2
(MANSP)NAT2
(NOMGA)NAT2
(TRACR)NAT2
nmol CoA per mg protein
min
0
5000
10000
15000
20000
0 1 2 3 4 5 6
0 1 2 3 4 5 6 7 8 9 10 11
0 1 2 3 4 5 6 7 8
0 2 4 6 8 10 12 14 16
PANS
PABA
INH
PA
5AS

Panel 3

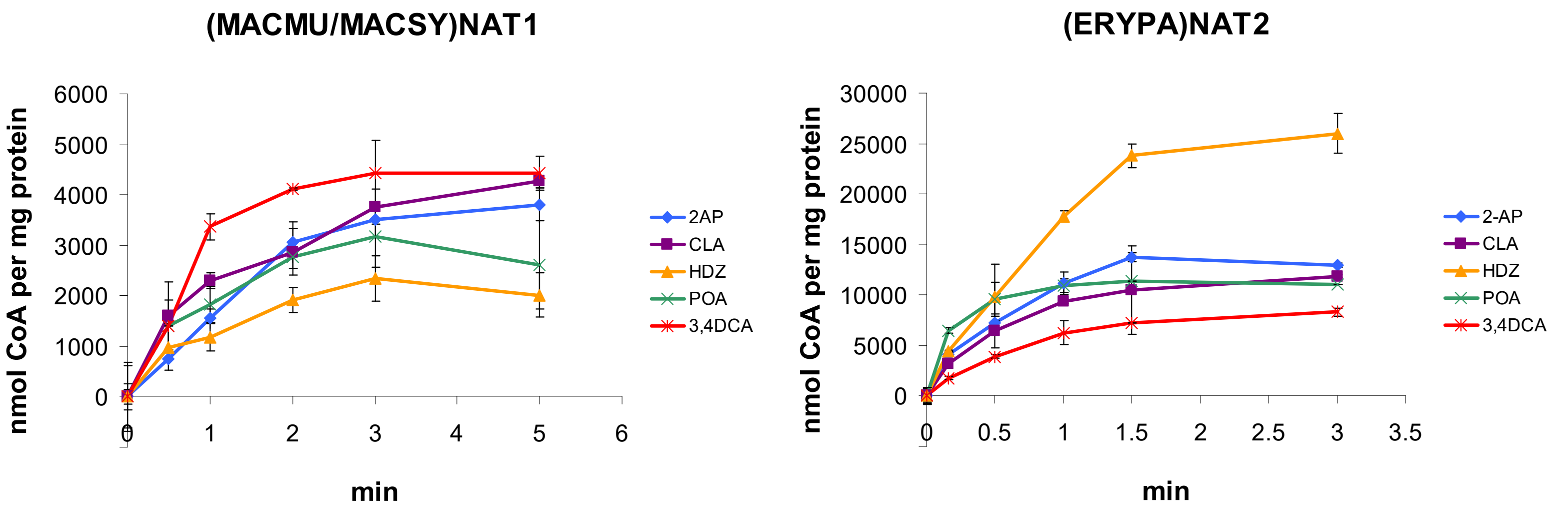

Panel 4

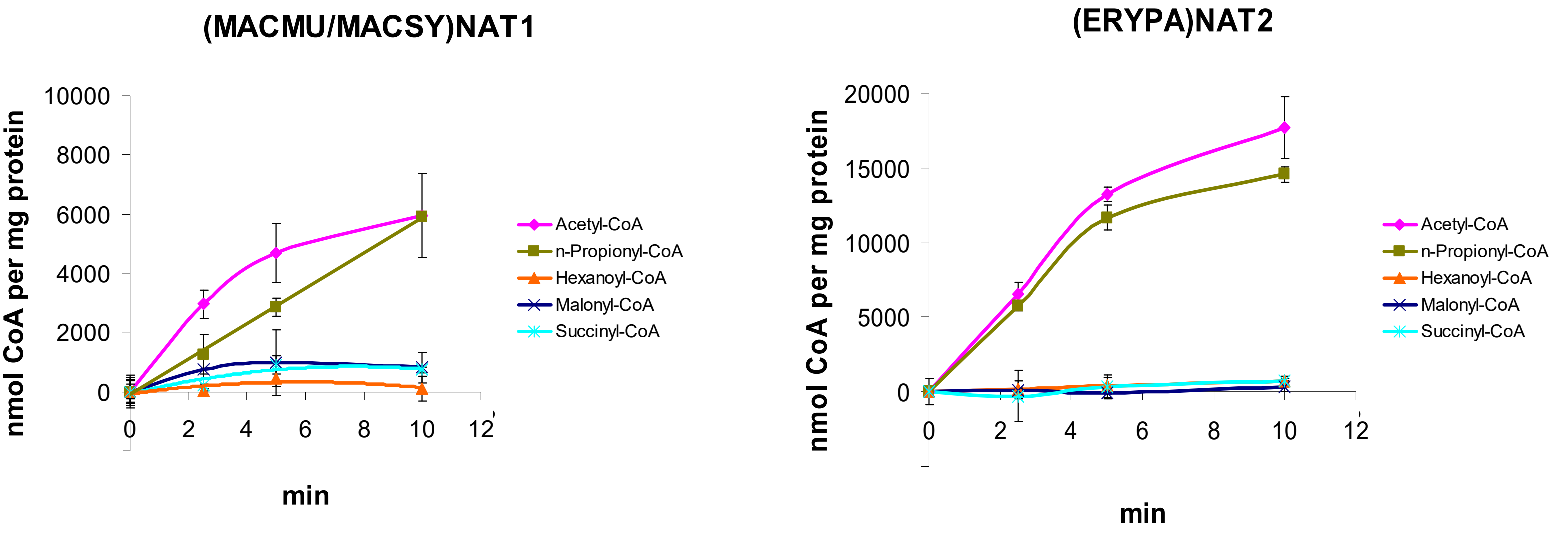

Expanded Data Supplementary Figure 2: Primate NAT proteins assayed for enzymatic activity with various substrates.

Enzymatic release of coenzyme A (CoA) was monitored with Ellman's reagent over specific time points, in reactions containing each purified protein, plus 0.4 mM of donor substrate (typically acetyl-CoA, unless otherwise indicated) and 0.5 mM of acceptor substrate (as indicated). Duplicate (Panels 1-3) or triplicate (Panel 4) reactions were performed and each data point is the average±standard deviation.

Panels 1 and 2 show assays with various NAT1 and NAT2 homologues. Panel 3 shows assays with additional acceptor substrates, using only the (MACMU/MACSY)NAT1 and (ERYPA)NAT2 homologues. Panel 4 shows assays of (MACMU/MACSY)NAT1 and (ERYPA)NAT2 against five different donor substrates, and the acceptor substrates were PABA and PANS, respectively.
